# Fruit Water Stress Index of Apple Measured by Means of Temperature-Annotated 3D Point Cloud

**DOI:** 10.34133/plantphenomics.0252

**Published:** 2024-09-18

**Authors:** Nikos Tsoulias, Arash Khosravi, Werner B. Herppich, Manuela Zude-Sasse

**Affiliations:** ^1^ Leibniz Institute for Agricultural Engineering and Bioeconomy (ATB), Department Agromechatronic, WG Precision Horticulture, Potsdam, Germany.; ^2^ Department of Agricultural, Food and Environmental Science, Marche Polytechnic University, 60131 Ancona, Italy.

## Abstract

In applied ecophysiological studies related to global warming and water scarcity, the water status of fruit is of increasing importance in the context of fresh food production. In the present work, a fruit water stress index (*FWSI*) is introduced for close analysis of the relationship between fruit and air temperatures. A sensor system consisting of light detection and ranging (LiDAR) sensor and thermal camera was employed to remotely analyze apple trees (*Malus* x *domestica* Borkh. “Gala”) by means of 3D point clouds. After geometric calibration of the sensor system, the temperature values were assigned in the corresponding 3D point cloud to reconstruct a thermal point cloud of the entire canopy. The annotated points belonging to the fruit were segmented, providing annotated fruit point clouds. Such estimated 3D distribution of fruit surface temperature (*T*_Est_) was highly correlated to manually recorded reference temperature (*r*^2^ = 0.93). As methodological innovation, based on *T*_Est_, the fruit water stress index (*FWSI*_Est_) was introduced, potentially providing more detailed information on the fruit compared to the crop water stress index of whole canopy obtained from established 2D thermal imaging. *FWSI*_Est_ showed low error when compared to manual reference data. Considering in total 302 apples, *FWSI*_Est_ increased during the season. Additional diel measurements on 50 apples, each at 6 measurements per day (in total 600 apples), were performed in the commercial harvest window. *FWSI*_Est_ calculated with air temperature plus 5 °C appeared as diel hysteresis. Such diurnal changes of *FWSI*_Est_ and those throughout fruit development provide a new ecophysiological tool aimed at 3D spatiotemporal fruit analysis and particularly more efficient, capturing more samples, insight in the specific requests of crop management.

## Introduction

Due to increasing awareness of global warming-related water scarcity, water status analysis has been addressed as pivotal topic in ecophysiological studies capturing also woody plants in fruit production [1]. The temperature and water status of fruit tree is closely related to orchard yield performance [2–4]. Moreover, high fruit temperature can lead to fruit damage appearing more frequently due to climate change and resulting increase in the maximum temperature as well as appearance of heat waves [5]. Consequently, suitable methods for monitoring the crop water status are essential for ecophysiological studies and resilient food production. Current monitoring systems can be defined according to requirements on the spatial and temporal resolution of applications, sensor platforms, and sensor types [6]. For monitoring of tree and fruit water status, proximal and remote sensors can be applied. Proximal sensors can be used in individual trees [7,8], but the representation of the individual response of fruit, trees, and group of trees depends on the homogeneity of the orchard [9]. Considering older orchards or enhanced biodiversity within an orchard, the representation capacity of few individual trees and fruit data is certainly low. Consequently, various sensor types and their combinations are necessary to obtain comprehensive data [10]. In this context, e.g., various combinations of fruit gauges and leaf patch clamp pressure probes have been employed for continuous monitoring of olive (*Olea europaea* L.) and nectarine (*Prunus persica* L.) addressing the tree water status [10–12]. Apple trees (*Malus* x *domestica* Borkh.) were monitored by both fruit gauges and Scholander pressure chambers [13], while Grilo et al. [14] continuously applied a combination of fruit gauges and sap flow sensors on orange trees (*Citrus sinensis*). Although these combinations of proximal sensors increased accuracy, the practical use of this approach is limited by the variability within a large number of trees, complex specific installation and maintenance of sensors, and the correct interpretation of the various water status parameters under highly variable experimental or commercial orchard conditions [10,15,16].

Remote sensing at the fruit level may overcome these drawbacks, since the sensors capture many or even all trees in the orchard. Noninvasive techniques, including 2-dimensional (2D) machine vision systems combining 2D color or spectral data in short-wave up to thermal infrared wavelength ranges, have shown promising results in agriculture. Particularly, the crop water stress index (*CWSI*) has been widely used as a remote sensing method for water-deficit stress detection of entire canopies by means of thermal imaging [17,18]. *CWSI* is interesting due to the close relationship between canopy transpiration rate and surface temperature [19,20]. *CWSI* has, e.g., been used for large-scale monitoring via airborne systems, which made it suitable for real scenario application in commercial orchards [21]. On-the-ground and airborne canopy temperature measurements evaluated water deficits in peach trees under different irrigation strategies by means of *CWSI* [22–24]. *CWSI* calculated from both approaches was highly correlated with stem water potential, while canopy temperature closely reflected stomatal conductance. Most orchards, however, are too small for satellite measurements and provide mixed pixels of plant rows and ways disabling information gaining on the canopy. Therefore, unmanned aerial vehicles (UAVs) and ground-based thermal imaging platforms have been widely used for crop water-deficit stress detection [25,26]. Among others, *CWSI* was applied in peach to monitor irrigation requirements and identify areas sensitive to water deficit under low-frequency irrigation systems [24,27,28].

For various tree crops, studies investigated the correlation between *CWSI* and plant physiological water-deficit stress parameters such as leaf and/or stem water potential and stomatal conductance or transpiration rate [22,29–33]. *CWSI* is inversely correlated to stem [34] and leaf water potential [27,35]. Moreover, leaf and canopy water status were investigated remotely by estimating *CWSI* from aerial thermal imagery in date-palm (*Phoenix dactylifera* L.) trees [36]. Imaging in optical red, green, and blue (RGB)ranges and thermal cameras were applied to assess the relationship between leaf water potential and *CWSI*, resulting in *r*^2^ of 0.67 and 0.47, respectively, in the shaded and the sunlit side of the canopy [37]. RGB imaging and infrared thermography were applied to evaluate *CWSI* of pistachio (*Pistacia vera* L.) trees using convolutional neural networks [38]. UAV-based thermal and multispectral images were employed to analyze *CWSI* and normalized difference vegetation index (*NDVI*) in mild and moderately stressed almond [*Prunus dulcis* Mill. (D.A. Web)] trees [39].

*CWSI* has been calculated based on empirical [40] or theoretical [41] approaches. Recently, these equations were developed further [20,42]. However, analysis of canopy *CWSI* is based on the water deficit-induced reduction of stomatal conductance and, thus, transpiration of leaves, but assuming that the leaf water status closely reflects the fruit water status would be speculative. Up to now, an index similar to CWSI was never used to analyze the actual fruit water status.

Furthermore, spatial analysis is still lacking considering fruit data [43]. Modeling approaches were undertaken using RGB and thermal imaging to estimate the fruit temperature in 2D [44]. Easier to access weather data were applied to estimate the mean fruit surface temperature, resulting in reasonable performance of root mean square error (*RMSE*) <2 °C [45]. However, the spatial distribution of fruit temperature was challenging with this approach. In recent years, light detection and ranging (LiDAR) technology has been extensively used for remote sensing in arable farming and forestry, due to its ability to provide high-resolution 3D geometric information of plants in field conditions as 3D point clouds [46–48]. Terrestrial LiDAR sensors have been employed to develop estimation methods for geometric fruit parameters [49,50] as well as to map flowers and fruit in almond orchards [51], Kang et al., 2022. The advancement in terrestrial LiDAR sensors has also enabled the acquisition of the intensity of backscattered reflection at each point measured. This provides additional information for the segmentation of fruit from the 3D point cloud of whole canopy [49,52] and for the quantification of chlorophyll content of individual fruit [53,54]. Combining LiDAR and thermal imaging is proposed in this study for providing geometric and thermal data for innovative 3D tree analysis at the fruit level.

In other disciplines such as architecture and robotics [55], LiDAR-based 3D point clouds have been annotated with temperature data from thermal cameras. Having the 3D fruit visualization by LiDAR at hand, the annotation with temperature data enables to analyze 4D point clouds of canopies and fruit. Recently, a terrestrial LiDAR laser scanner was coupled with a thermal camera for reconstructing the 3D thermal point cloud in avocados and apples [56,57]. In basic ecophysiological studies capturing the variation of growth factors occurring due to global warming, such plant phenotyping is a modern technology to capture data from many individual plants. While root phenotyping [58,59] and remote sensing of whole canopies [60] is well established, the temperature of fruit surfaces (FST) was rarely tackled [45]. However, FST represents an important variable when discussing fruit growth and risk of fruit damage. Particularly, the 3D distribution within the canopies would be valuable for precise crop management. Furthermore, from the 3D thermal point clouds, a new fruit water stress index (*FWSI*) could be derived, providing an informative, spatiotemporally resolved parameter for analyzing fruit.

Therefore, the objectives of the present study were (a) to derive and validate the fruit surface temperature in 3D from terrestrial mobile LiDAR sensor and thermal imaging, (b) to calculate and compare estimated and manually measured *FWSI*, and (c) to gain first results with the new method on the course of *FWSI* changes during the growth season.

## Materials and Methods

### Experimental layout

The experiment was conducted in the Field Lab for Digital Agriculture of Leibniz Institute for Agricultural Engineering and Bioeconomy (ATB), located in Potsdam-Marquardt, Germany (latitude: 52.466°N, longitude: 12.572°E), planted with *Malus* × *domestica* Borkh. “Gala-Brookfield” on M9 rootstock with 0.95-m distance between trees, trained as slender spindle with an average tree height of 2.8 m. Trees were statically supported by horizontally parallel wires. Measurements took place on 7 trees throughout the season midday at 67, 81, and 132 days after full bloom (*DAFB*_67_, *DAFB*_81_, and *DAFB*_132_, respectively) (Table 1). Diurnal measurements were carried out on 5 trees at *DAFB*_152_ and *DAFB*_153_ at 0700, 0800, 1000, 1200, 1300, and 1800, and *DAFB*_166_. However, measurements on *DAFB*_166_, collected at midday (*n* = 50), have been used exclusively to complete the seasonal fruit trend analysis (Table 1).

**Table 1.** Variable (Var) measured during seasonal fruit development or within 2 consecutive days as diel dataset with number of trees (*Tree*), number of samples per tree (*Sample*), and frequency (*f*) according to date in days after full bloom (*DAFB*) and total number of samples (*n*).

Var	Period	Descript.	Number									*n*
*T_Ref_*	Season	*DAFB*	53	67	81			132			166	
		*Tree*		7	7			7			5	
		*Sample*		6	6			6			10	
		*f*		2	2			2			1	302
	Diel	*DAFB*							152	153		
		*Tree*							5	5		
		*Sample*							10	10		600
		*f*							6	6		(300 per day)
*T_Leaf_* *g_st_*	Season	*DAFB*	53	67	74	88	109	117	123	151	165	
		*Tree*	6	6	6	6	6	6	6	6	6	138
		*Sample*	3	3	3	3	3	3	2	1	2	
		*f*	1	1	1	1	1	1	1	1	1	
*T_Leaf_* *Ψ_Leaf_*	Diel	*DAFB*							152	153		
		*Tree*							5	5		
		*Sample*							3	3		150
		*f*							5	5		(75 per day)
*Ψ_Leaf_* *Ψ_Stem_*	Season	*DAFB*		61	68	81	110		124			
		*Tree*		3	3	3	3		3			30
		*Sample*		2	2	2	2		2			
		*f*		1	1	1	1		1			
Fruit quality	Season	*DAFB*		67		81		132			166	
		*Tree*		4		4		4			5	34
		*Sample*		2		2		2			2	
		*f*		1		1		1			1	

Further manual measurements were carried out on the trees (Table 1). Weather data (Uniklima Vario, Toss, Potsdam, Germany) were obtained in the orchard at 15 -min intervals, recording air temperature (°C), relative air humidity (%), global normal irradiance (*GNI*; W m^−2^), and precipitation (mm). The water vapor partial pressure deficit (*VPD*, kPa) was calculated [61] as:

$$\mathrm{VPD}=\left(1-\left(\mathrm{RH}/100\right)\right)\times \mathrm{SVP}$$ (1)

$$\mathrm{with}\;\mathrm{SVP}\;\left(\mathrm{PA}\right)=610.7\times 10^{\left(7.5T/\left(237.3+T\right)\right)}$$ (2)

where *RH* is the relative humidity, *SVP* is the saturated vapor pressure, and *T* is the temperature (°C).

### Remote sensing

The phenotyping system was set up on a circular conveyor platform in the experimental apple orchard after calibrations of sensor signal intensity and position. The platform used an electrical engine (DRN71, SEW Eurodrive, Germany) operating at 50 Hz and a stainless-steel chain with mechanical suspensions to support the plant sensors. A mobile 2D LiDAR sensor emitting at a wavelength of 905 nm (LMS-511, Sick AG, Waldkirch, Germany) was mounted vertically on a metal plate placed at one suspension at 0.7 m above the ground level. The LiDAR sensor had the configuration with 0.1667° angular resolution, 25-Hz scanning frequency, and 180° scanning angle. The data were georeferenced using a real-time kinematic global navigation satellite system (AgGPS 542, Trimble, Sunnyvale, CA, USA), while orientation information was acquired using an inertial measurement unit (MTi-G-710, XSENS, Enschede, Netherlands), both of which were arranged on the sensor metal plate. The orientation information had an *RMSE* of 0.25° for roll, pitch, and yaw according to the manufacturer.

Additionally, a thermal camera (A655sc, FLIR Systems Inc., MA, USA) was installed, positioned 0.2 m above the LiDAR sensor at the same suspension. The camera provided the spatial resolution of 640 × 480 pixels at 50 Hz and a spectral range from 7.5 to 14 μm, with an operational temperature range from −40 °C to 150 °C and a thermal resolution of <0.05 °C. A lens (T198065, Teledyne FLIR LLC, Wilsonville, USA) with focal length of 6.5 mm (diagonal 80^o^) was attached to the camera. The LiDAR sensor and thermal camera underwent intrinsic and extrinsic calibrations using a lightbulb pattern [57] for determining the geometric calibration of each sensor and of both sensors in relation to each other. Points from LiDAR sensor captured also with thermal camera were annotated with thermal information (°C), resulting in spatially resolved *T*_Raw_ data of canopies. All raw data are available open access [62].

### LiDAR data processing for tree and fruit segmentation

The apparent return signal strength intensity (*R*_ToF_) was recorded at 905 nm for each point in the 3D point cloud. Calibration algorithm for *R*_ToF_ was obtained with checker board coated with white powder (BaSO4, CAS number: 7727–43-7, Merck, Darmstadt, Germany), serving as 100% reference and black staining (S black, Avian Technologies, New London, USA) for 0% reference [53].

The 3D point cloud data were generated in Matlab environment (Computer Vision Toolbox, 2018b, Mathworks, Natick, USA) for positioning and alignment of 2 tree sides, for the latter using iterative closest point (ICP) algorithm, as described earlier [63]. To obtain points per tree (*PPT*), trees were segmented based on their stem position and planting distance. The maximum laser points in bivariate point density histogram were assumed to represent the center of the canopy. This assumption was made for the present slender spindle tree training system. For each tree point cloud, the volume of cylinder with a diameter of 0.95 m around the estimated stem position coordinates was considered.

Position and shape of apples within the tree point clouds were determined based on the geometric features, linearity (*L*), curvature (*C*), and *R*_ToF_, considering each point of the 3D tree point cloud [49]. The temperature information was not considered in the segmentation. In the 3D point clouds of trees, local neighbors were decomposed and eigenvalues were calculated (*λ*_1_, *λ*_2_, *λ*_3_). To distinguish the 3D points of woody parts (*W*) from leaves and fruit, the probability density function was used to gain the mode value of the distribution of *L*_W_, *C*_W_, and *R*_ToF,W_, which were used as thresholds (*R*_th,W_, *C*_th,W_, and *L*_th,W_). Points that met the criteria of *L*_W_ ≤ *L*_th,W_, *C*_th,W_ ≤ *C*_W_, and *R*_th,W_ ≤ *R*_ToF,W_ were segmented and subtracted from the total number of *PPT*. Similarly, threshold values of apple points in terms of C and reflected intensity (*C*_A_ and *R*_ToF_,_A_) were used. For example, *C* values closer to 100 indicated a higher likelihood for the shape of neighboring point’s appearance to be curved. Neighboring points, which met the criteria of *C*_th,A_ ≤ *C*_A_, and *R*_th,A_ ≤ *R*_ToF,A_, were segmented and annotated as apple. Subsequently, a density-based scan algorithm (DBSCAN) was applied (a) to find point sets with the assumed diameter (obtained as mean value from manually measured diameter of fruit) found in the neighborhood and (b) the value 10 (based on preliminary results as the minimum number of neighbors). The mean surface temperature values of segmented apples (*T*_Raw_) were considered in the further calculation. All annotated point clouds are available open access [62].

### Reference temperature

During the period of fruit development, the surface temperature of apples [7 trees × 6 fruit × 2 (left and right side of each fruit) × 3 dates results in *n* = 252] was manually measured (*T*_Ref_) with an infrared thermometer (Microscanner D501, Exergen, Watertown, USA). Similarly, during the diurnal temperature measurements of *T*_Ref_, apples (*n* = 50) were measured 6 times (0700, 0800, 1000, 1200, 1300, and 1800) data capturing 300 readings on each of the 2 consecutive days.

*T*_Ref_ was compared to the corresponding temperature *T*_Raw_ derived from remote sensing approach. All datasets obtained over the season in the early afternoon (dataset from seasonal readings plus 50 fruits from diurnal course at 1300 on September 21) were used for building the calibration of fruit surface temperature and its cross-validation. For this purpose, the dataset (*n* = 302) was split in 80% (*n* = 241) for calibration and 20% (*n* = 61) for cross-validation in block-wise design for each measuring date.

To determine the local maximum (^max^*T*) and minimum (^min^*T*) temperatures among the apples, 2 individual fruits were covered with Vaseline and soap mixed with water, respectively. Their fruit surface temperature was additionally measured as *T*_Raw_ and *T*_Ref_.

### *FWSI* estimation

Temperature points of fruit surfaces obtained by means of temperature-annotated LiDAR point cloud were exploited to estimate *FWSI* by 3 alternative methods (Eqs. 3, 5, and 6).

*FWSI_I_* was calculated according to the approach by Irmak et al. [64]:

FWSIITEst−TminEstTa+5−TminEst (3)

where *T*_Est_ represents the temperature for each point of apple surface and ^min^*T*_Est_ is the minimum fruit temperature of segmented point clouds, measured at each individual measuring day. *T*_a_ is the average air temperature (Eq. 4) plus 5 °C as proposed by Irmak et al. [64].

$$Ta=\frac{\sum_{n-5}^{n-2}\;T_{i}}{4}$$ (4)

where *T_i_* is the mean air temperature in the time range *n* being the exact hour of measuring fruit temperature at each individual measuring day.

*FWSI*_J_ was calculated according to [65]:

FWSIJ=TEst−TWEstTDEst−TWEst (5)

where ^W^*T* is the temperature of fully transpiring fruit and ^D^*T* is the temperature of nontranspiring, Vaseline-coated fruit measured at each individual measuring day.

*FWSI*_N_, called normalized fruit water stress index, was calculated as:

FWSIN=TEst−TminEstTmaxEst−TminEst (6)

^min^*T*_Est_ and ^max^*T*_Est_ represent the minimum and maximum fruit temperature of the segmented point clouds, measured at each individual measuring day.

For gaining an insight in the performance of different *FWSI* approaches, ∆*T* (°C) has been employed according to Eq. 7.

$$∆T=T_{\text{Est}}-T_{a}$$ (7)

where both actual fruit (*T*_Est_) and air temperature *T*_a_ (Eq. 4) were considered.

For calculating the error of the method, for each of the 3 approaches, either *T*_Est_ from temperature-annotated 3D point cloud was inserted as stated in Eqs. 3 to 6 or the manually measured reference fruit surface temperature *T*_Ref_ was used.

### Water potential

Leaf and stem water potential (*Ψ*_stem_) were measured on 5 neighbor trees with a portable Scholander pressure chamber (Plant Water Status Console 3000, Soilmoisture Equipment Corp., Pullman, USA) capturing direct readings or shaded leaves (*n* = 3) sealed in plastic bags for 30 min. The temperature from 3 neighbor leaves was manually measured (*T*_Leaf_) during each *Ψ*_Stem_ reading. Measurements were carried out on DAFB_152_ and DAFB_153_ at 0700, 0900, 1100, 1300, and 1700. An average of 4 measurements was taken for each individual tree sampled (*n* = 75; Table 1).

### Dendrometer and leaf gas exchange

The maximum daily shrinkage of tree stem was recorded using dendrometers (DD-L, Ecomatic GmbH, Dachau, Germany), installed 70 cm above ground, 50 cm above tree’s grafting zone. Data were recorded with data logger (CR10X and AM416 multiplexer, Campbell Scientific, Logan, USA).

Light response of steady-state leaf gas exchange was measured on 3 mature leaves selected at bearing shoots, of 3 neighbor trees from other trees subjected to LiDAR analysis, with a portable gas exchange analyzer (LI-6400 XT with LI-6400-40 red/blue LED, LI-COR Inc., Lincoln, USA). At ambient leaf temperature, relative humidity, and constant CO_2_ mole fraction in the reference gas (400 μmol mol^−1^), analyses were performed at photosynthetic photon flux rate (*PPFR*) of 2,000, 250, 100, 50, 20, and 0 μmol m^−2^ s^−1^ with a minimum waiting time of 100 s before each measurement. Maximum quantum yield (^max^*α*, mol mol^−1^) and the rate of light-saturated CO_2_ gas exchange (^max^*J*_CO2_, μmol m^−2^ s^−1^) were considered [16].

### Fruit quality

After each measuring date with sensor system, apple samples (*n* = 8) were collected for fruit characterization in the laboratory. Fruit diameter (*D*) was manually measured by means of a digital caliper gauge considering the mean diameter of 2 measurements taken equatorially with 90^o^ difference. The resulting mean diameter was applied in fruit segmentation process. Fresh mass (*FM*) was measured gravimetrically. Soluble solid content (*SSC*) was measured on squeezed juice obtained during firmness test, collected with pipette, and analyzed by refractometry (Pal-1, Atago, Tokyo, Japan). Stress and strain curves were measured by Texture Analyzer (TA-XT Plus, Stable Micro Systems, Godalming, UK) using a convex probe with a diameter of 11.1 mm, at constant speed of 4 mm s^−1^ on a peeled area in the equatorial region. Fruit flesh firmness was analyzed as the force obtained at 8-mm depth of penetration, corrected by probe size.

The chlorophyll content of apple skin and hypodermis (2 mm thickness), capturing contents of chlorophyll_a and _b, and pheophytin_a, was analyzed after acetone/diethyl ether extraction [66]. Extracts were filtered using a glass frit (pore size 3) attached to a vacuum pump, and phase separation was carried out in separating funnel by adding distilled water, transferring nonpolar pigments such as chlorophylls to nonpolar diethyl ether phase. The absorbance spectrum of the nonpolar phase was recorded spectrophotometrically (Lambda 950, Perkin Elmer Inc., Waltham, USA). The standard spectra of 3 chlorophylls were considered in the iterative multiple linear regression spectral analysis [67].

### Statistical analysis

Descriptive statistics were applied to all datasets capturing minimum, maximum, mean, and standard deviation. A regression analysis was performed to quantify linear and logarithmic relationships between the manual measurements and LiDAR-derived data over the growing stages, and *RMSE*, mean bias error, and coefficient of determination (*r*^2^) were calculated. Descriptive statistics and graph design were performed using Sigmaplot 14.5 (Systat Software Inc., San Jose, USA).

## Results and Discussion

### Fruit surface temperature estimation

**Fig. 1.**
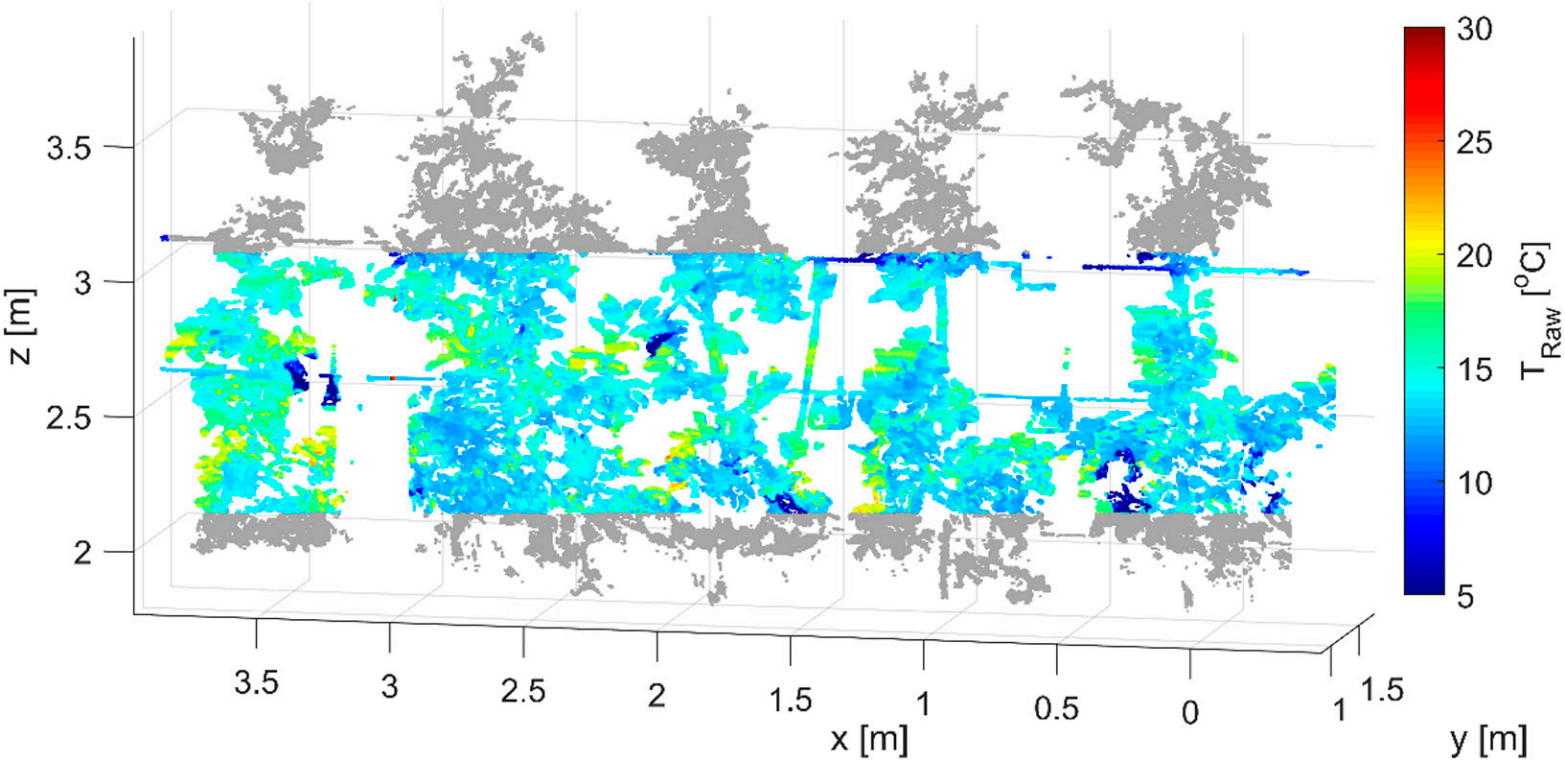
3D spatial temperature distribution in the canopy analyzed by means of LiDAR and thermal sensors at *DAFB*_153_.

Slender spindles form the major training system of apple trees in worldwide production, providing a 3D structure in which the fruits are more or less evenly distributed according to the success of the crop load management. Apple surface temperature was monitored employing the LiDAR sensor to gain geometric position data in the format of 3D point cloud of the trees. Furthermore, a thermal camera was used to annotate the points with temperature information. The extrinsic geometric calibration was applied to acquire the thermal 3D point cloud of trees (Fig. 1), revealing an *RMSE* of 1.82% pixel^−1^. The estimated temperature (*T*_Raw_) in the annotated 3D point cloud of the trees varied (Fig. 1), with lower temperatures in the upper parts of canopies at all measuring dates. Mean of *T*_Raw_ of stem points considering all measuring dates was 20.6 ± 0.65 °C standard deviation. The segmented leaf area showed a mean value of *T*_Raw_ = 18.3 ± 1.4 °C.

It was often assumed that fruit surface *T* is equal to air *T*, which was found to be untrue when analyzing the spatial distribution of apple temperature by means of few thermocouple readings and modeling the fruit temperature [68]. In the present study, application of the fruit segmentation routine [49] allowed to exclude leaf area and wood structure and segment the temperature-annotated point clouds of all fruit in the present study. The LiDAR system was used as a pilot sensor to segment the fruit point clouds from the overall canopy point clouds, revealing F1 scores of 80.7, 85.6, 90.2, and 91.4 for the measuring dates at *DAFB*_67_, *DAFB*_81_, *DAFB*_132_, and *DAFB*_166_, respectively. The bigger fruit at later growth stages provided better results as expected according to previous results [49]. The segmented, annotated fruit point clouds (Fig. S2) obtained were processed further as temperature means per fruit.

**Fig. 2.**
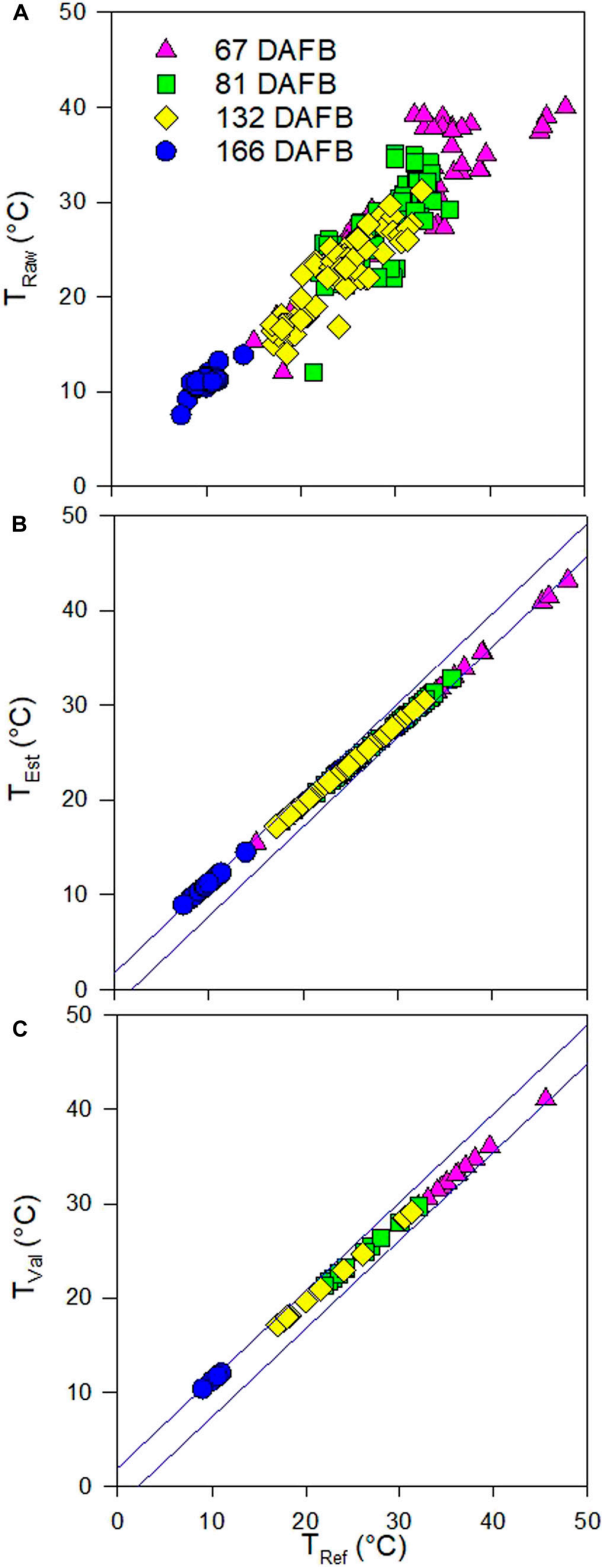
Scatterplots of (A) raw LiDAR-derived temperature (*T*_Raw_) (*n* = 302), (B) estimated from linear regression (*n* = 241) (*T*_Est_) and (C) cross-validation results (*T*_Val_) (*n* = 61). All measuring dates were included and marked in different colors.

Throughout the seasonal measurements, fruit temperature estimated by means of *T*-annotated 3D point clouds (*T*_Raw_) ranged from 7.55 to 40 °C with a mean of 23.4 °C, while *T*_Ref_ measured manually on apple surfaces varied between 8.94 and 43.18 °C. Considering all measuring days, mean *T*_Ref_ was 24.3 °C. *T*_Raw_ revealed the highest mean value of 25.41 ± 5.34 °C at *DAFB*_67_ beginning of July and the lowest mean value of 11.14 ± 0.94 °C at *DAFB*_166_ after harvest. For the same dates, *T*_Ref_ showed slightly enhanced values of 31.85 ± 2.55 °C and 11.43 ± 0.60 °C, respectively. Moreover, *T*_Raw_ depicted lower minimum temperatures on *DAFB*_67_ (15.42 °C) and *DAFB*_166_ (8.94 °C), in contrast to reference measurements 24.67 and 10.37 °C, respectively. Similarly, *T*_Raw_ measurements noted enhanced maximum values found between *T*_Raw_ and *T*_Ref_, revealing 43.18 and 34.76 °C at *DAFB*_67_ and 14.49 and 12.05 °C at *DAFB*_166_, indicating potential overestimation. However, the 2 variables were highly and linearly correlated (*r*^2^ = 0.91; *RMSE* = 2.54%; Table 2 and Fig. 2).

**Table 2.** Descriptive statistics of the relationship between fruit temperatures estimated from LiDAR and thermal sensors (*T*_Raw_) data and those measured manually (*T*_Ref_). Given are the coefficients of determination for calibration (*Τ*_Est_) and cross-validation (*T*_Val_), root mean square error (*RMSE*), and *bias*. For calibration, 80% of data (*n* = 241) were used, whereas the remaining 20% (*n* = 61) were analyzed in cross-validation.

	*Min*	*Max*	*Mean*	*Bias* (°C)	*r* ^2^	*RMSE* (%)
*T*_Ref_	8.94	43.18	24.30			
*T*_Raw_	7.55	40.00	23.40	−0.77	0.91	2.54
*T*_Est_	8.94	43.18	22.87	−0.99	0.93	1.59
*T*_Val_	10.37	41.10	24.14	−1.23	1.00	1.91

*T*_Raw_ data were calibrated based on *T*_Ref_ using 80% of the fruit data to achieve *T*_Est_. The robustness of calibration was assessed by means of cross-validation considering 20% of fruit data. The measuring uncertainty of calibration was 1.59% with *r*^2^ = 0.93, which was confirmed by slightly enhanced coefficient of determination appearing in the smaller dataset used in the cross-validation. The mean bias error increased 0.24 °C and *RMSE* by 0.32 °C, comparing calibration (Fig. 2B) and cross-validation results (Table 2 and Fig. 2C). To further enhance the robustness and generalizability of these findings, future studies could benefit from increasing both the sample size and the number of measurement days. This expansion would allow for more comprehensive validation and spatial analysis across varying climate conditions.

Despite the high correlation, problems with sensor drift can lead to inaccurate temperature readings. However, the blockwise cross-validation, taking 80%/20% from each measuring date, showed no systematic error (Fig. 2). Environmental factors such as ambient conditions, including humidity, wind, and direct sunlight, and variations in surface emissivity, e.g., by free water on the surface, can affect sensor readings [69]. In the present study, again, the errors appeared without change in the bias over the season (Table 2 and Fig. 2). More importantly, manual temperature readings may have introduced errors, because the time gap between the remote and manual readings may have caused deviation. On the other hand, the measurements in remote mode may have caused issues. Occlusions, where parts of the fruit or relevant surfaces are blocked by leaves, branches, or other fruit, can result in incomplete data capture and contribute to errors in temperature estimation [52]. Furthermore, some pixels may point to the sky instead of the tree surface. Also, wind can affect the actual surface that is captured with both sensors. Finally, data processing algorithms and interpolation methods used to create 3D point clouds can also introduce errors if they do not accurately account for all variables [57].

### Comparison of fruit water stress index approaches

Based on *T*_Est_, alternative fruit water stress indices (*FWSI*) were obtained (Table 2) with established equations for calculating the crop water-deficit stress index of canopies based on 2D thermal imaging. Variation of *FWSI*_J_, calculated according to [65], was generally high, with *FWSI*_J,Est_ ranging from −2.81 to 6.06 and *FWSI*_J,Val_ from −1.69 to 5.39. The wet and dry references obviously failed in apple fruit. Similar results were found in leaves [70], showing that Vaseline may have different effects than simply hindering transpiration, whereas the soap solution frequently dried before the remote reading was finalized.

**Fig. 3.**
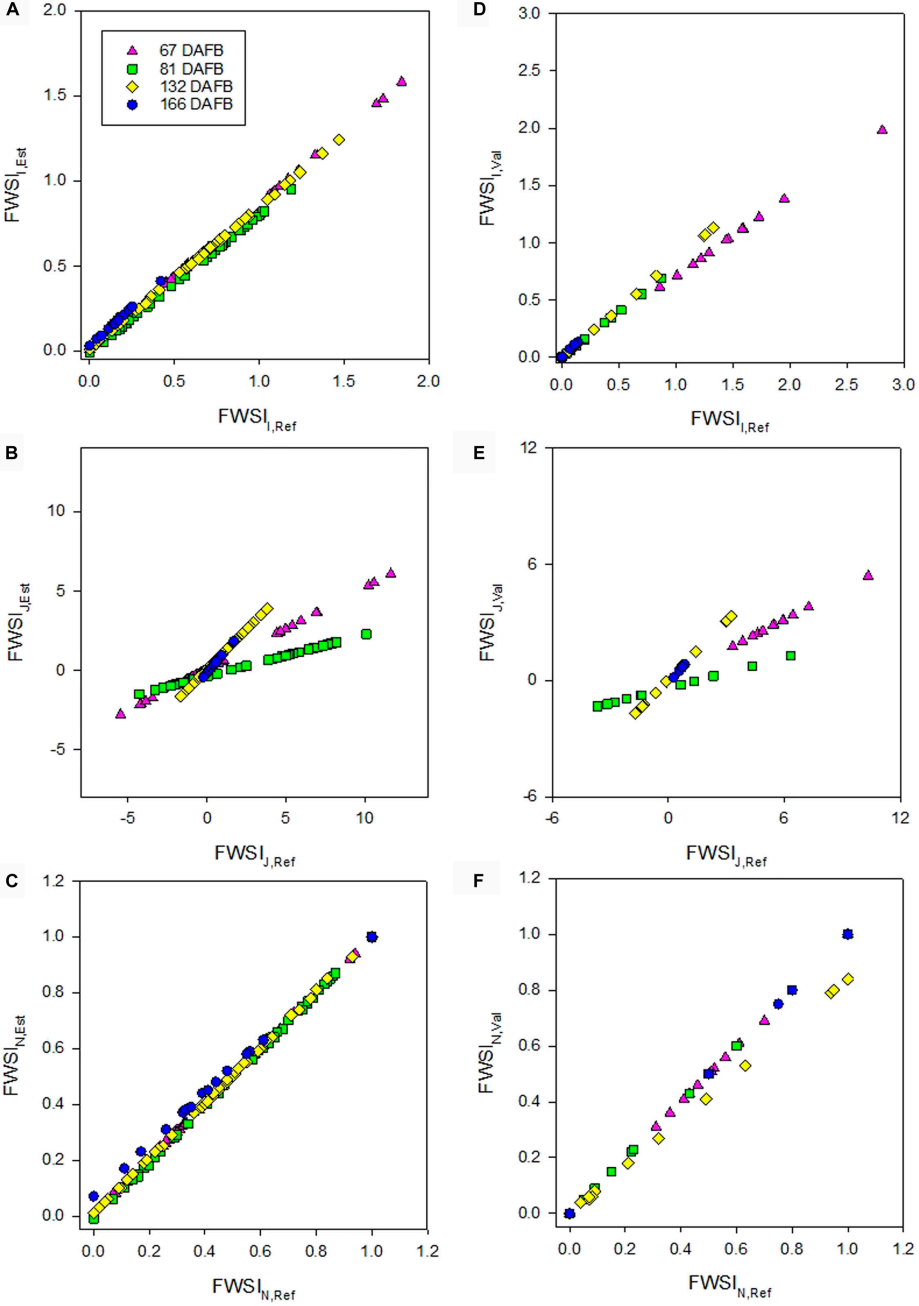
Fruit water stress index (*FWSI*) calculated by 3 approaches according to Jones [65], Irmak et al. [64], and normalized index considering a data split for calibrated fruit surface temperature data (80% of entire dataset, *n* = 241) shown in the left column (A to C) and results from the remaining 20% of dataset used for cross-validation *(n* = 61) in the right column (D to F).

Additionally, the coating may have affected the LiDAR readings, since the bias and *RMSE* were high when comparing *FWSI*_J_ based on remote sensing and based on manually recorded fruit temperature. Since the error of temperature estimation was low in untreated apples, such influence can be assumed. Accurately measuring of the temperature on a fully transpiring (^W^*T*) and nontranspiring (^D^*T*) apple surface may be an arduous challenge due to the need to maintain the correspondent status uniformly within the fruit surface. Similarly, applying Vaseline on vine leaves to estimate the water stress index have shown that the method is far from practical for large-scale agriculture [71]. This can potentially lead in lack of uniformity of water stress index not only within the tree canopy but also on the fruit surface during the growing stages. Artificial reference surfaces, such as green hemispherical cellulose surfaces, obtained high correlations with ^D^*T* and could potentially replace the coated Vaseline approach [72]. In the present study, measuring uncertainty was enhanced for *FWSI*_J,Est_, as indicated by *r*^2^ = 0.66 and *RMSE* = 2.27% (Table 3 and Fig. 3B and E). *RMSE* values for *FWSI*_I,Est_ and *FWSI*_I,Val_ (Fig. 3A, C, D, and F) were lower (0.11% and 0.24%, respectively). Therefore, *FWSI*_J,Est_ was not considered in the later steps of this study.

**Table 3.** Coefficient of determination (*r*^2^), root mean square error (*RMSE*, %), and bias (%) of fruit water stress indices calculated from remotely measured, calibrated fruit temperature data and manual fruit temperature readings for *FWSI*_Est_ considering the calibration dataset (*n* = 241) and for *FWSI*_Val_ capturing the dataset, not used in the calibration, but cross-validation (*n* = 61).

	*Min*	*Max*	*Mean*	*Bias* (%)	*RMSE* (%)	*r* ^2^
*FWSI*_I,Est_	−0.01	1.58	0.48	−0.08	0.11	0.99
*FWSI*_I,Val_	0.00	1.98	0.50	−0.16	0.24	0.99
*FWSI*_N,Est_	−0.01	1.00	0.44	0.01	0.02	0.99
*FWSI*_N,Val_	0.00	1.00	0.46	−0.02	0.05	0.98
*FWSI*_J,Est_	−2.81	6.06	0.53	−0.89	2.27	0.66
*FWSI*_J,Val_	−1.69	5.39	0.91	−1.02	2.00	0.80

### Seasonal course of *FWSI*

Throughout the season, maximum air *T* reached 43 °C, whereas minimum air *T* was 12.6 °C. Global radiation and *VPD* were maximal 600 W m^−2^ and 3.2 kPa, measured at DAFB_73_ and DAFB_86_, respectively. Heatwaves occurred between measuring days 2 and 3, *DAFB*_90_ and *DAFB*_110_, with maximum values of air *T* and global radiation higher than 40 °C and 500 W m^−2^, respectively (Fig. S1). Accordingly, *VPD* reached a peak of 3.5 kPa at *DAFB*_90_. Fruit development took place according to the typical increase of fresh mass and changes in soluble solid content, and decrease in fruit flesh firmness (Table S1).

**Fig. 4.**
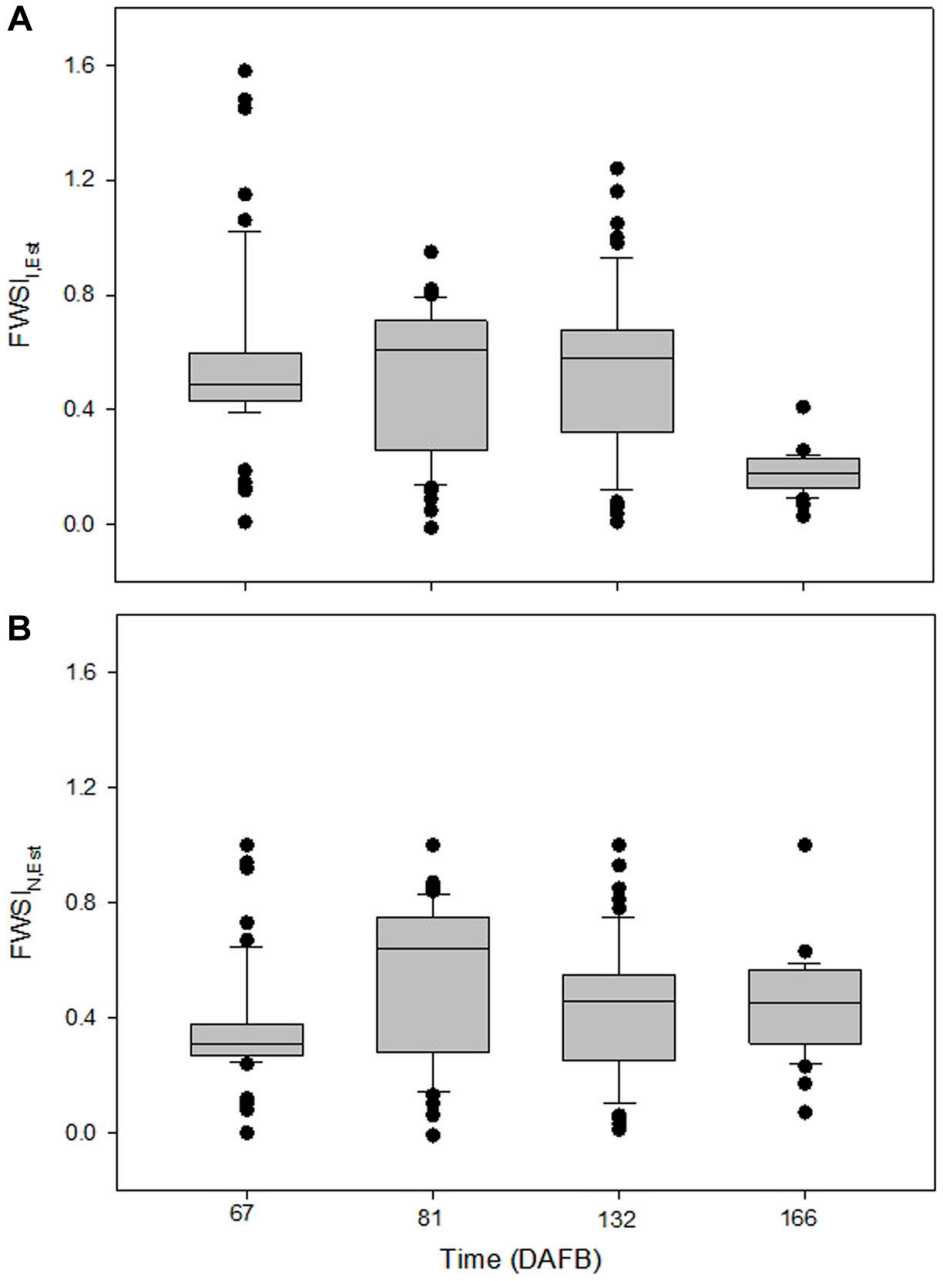
Box plot (A) of *FWSI*_I,Est_ as derived according to Irmak et al. [64] and (B) of the normalized *FWSI* (*FWSI*_N,Est_) calculated from measured local ^max^*T*_fruit_ and ^min^*T*_fruit_ in *T*_Est_ data of fruit considering 42 apples per measuring date, during fruit development in days after full bloom.

**Fig. 5.**
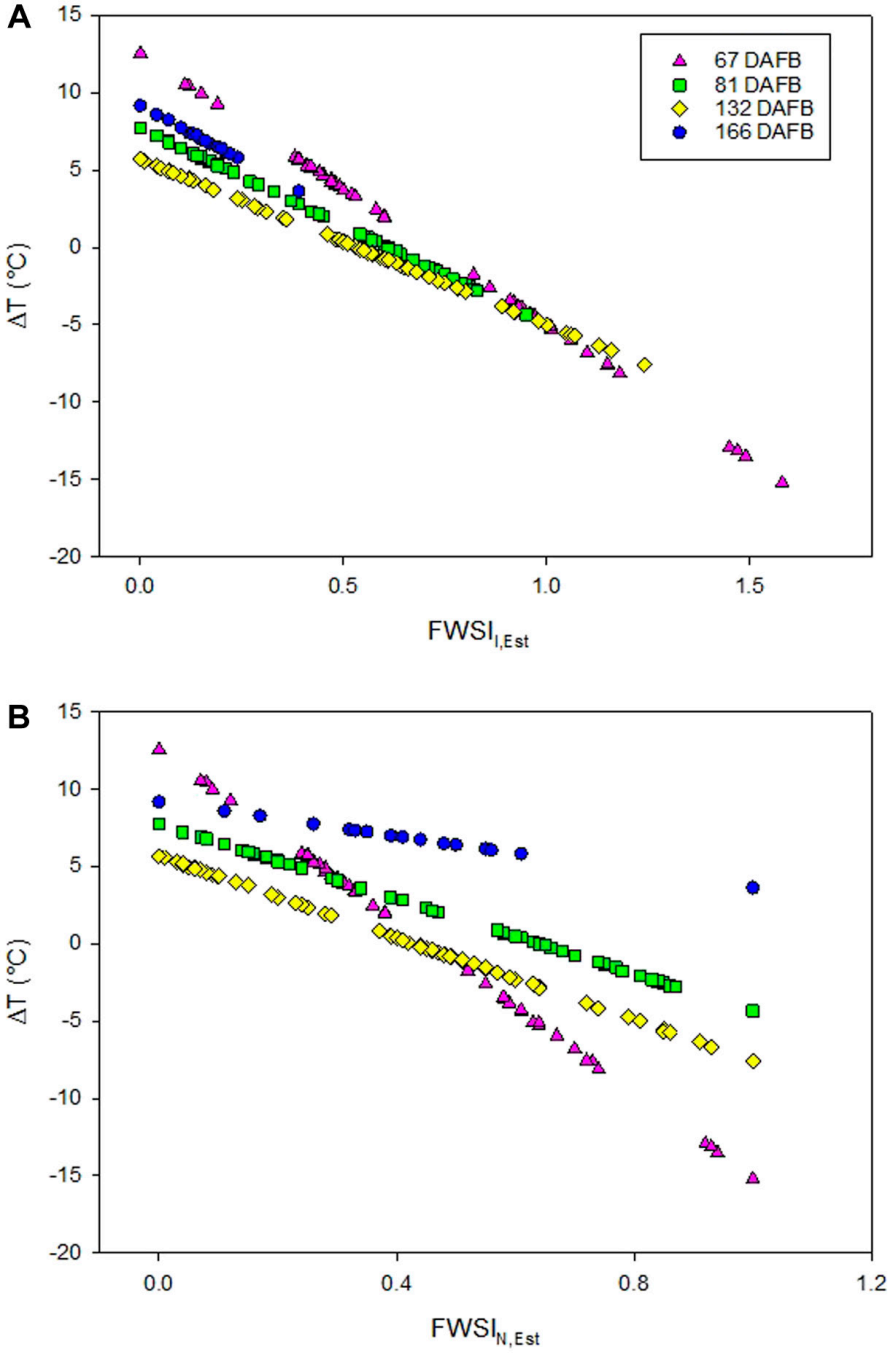
Relationships between the difference of air temperature and fruit surface temperature (Δ*Τ*) in relation to the fruit water stress indices (A) calculated according to Irmak et al. [64] with ^air^*T* plus 5 °C (*FWSI*_I,Est_) and (B) normalized on fruit surface temperature data (*FWSI*_N,Est_), analyzed on 4 days after full bloom (*DAFB*; 67, 81, 132, and 166) during fruit development.

Cross-validation of fruit surface temperature estimation (Table 1) confirmed that, after calibration, remote sensing data provided accurate *T* data of the fruit surface and can be applied in fruit monitoring. Thus, this approach was applied on all fruit data, remotely measured throughout the season 2022 considering measurements in the early afternoon. Subsequently, *FWSI_I_* according to approaches by Irmak et al. [64] and the normalized *FWSI_N_* were selected due to low measuring uncertainty when compared to the reference data. In comparison of both approaches, the mean *FWSI*_I,Est_ was slightly higher (0.52) than *FWSI*_N,Est_ (0.38) at *DAFB*_67_. Both indices showed marginal differences between *DAFB*_81_ and *DAFB*_132_. *FWSI*_I,Est_ showed decreased values of 0.62 ± 0.1 (mean ± SD) on *DAFB*_132_ to 0.18 ± 0.02 on *DAFB*_166_ (Fig. 4 and Fig. S2). On the other hand, variability of *FWSI*_N,Est_ was high on *DAFB*_166_, with similar means as on *DAFB*_132_. Specific impacts of development and physiological and physical properties of fruit may explain the distinct temporal variation of both indices. For example, the increased leaf temperature and reduced stomatal conductance, which was observed between *DAFB*_81_ and *DAFB*_132_, may have resulted in the increased *FWSI* (Fig. S5). In contrast to *FWSI*_N,Est_, *FWSI*_I,Est_ illustrated a similar pattern with the leaf temperature and higher variation due to the consideration of *T*a in the equation. Therefore, *FWSI*_I,Est_ decreased during fruit development (Fig. 5A).

On *DAFB*_67_, Δ*Τ* was high, as indicated by ^max^*T* = 14.1 °C and ^min^*T* = −14.8 °C (Fig. 5). Values of *FWSI*_I,Est_ remained unchanged *DAFB*_81_ and *DAFB*_132_. The range of Δ*Τ* was reduced on *DAFB*_166_, varying from 5.0 to 9.8 °C. *FWSI*_I,Est_ (Fig. 4A) may be dominated by the difference of air temperature and *FWSI*_I,Est_ as shown earlier for *CWSI* considering leaves [73]. Consequently, *FWSI*_I,Est_ may reflect the impact of high air *T* and possibly risk of heat damage and/or need for irrigation water. However, more work is requested to evaluate FWSI in specific experiments. Considering the present seasonal data, in temperate climate, the correlation between Δ*Τ* and *FWSI*_I,Est_ points to a close relationship from *DAFB*_67_ to *DAFB*_166_ (Fig. 5A). Similar correlation revealed that with *FWSI*_Ν,Est_ (Fig. 5B), however, the overall correlation was slightly weaker with 0.95 and 0.79 for *FWSI*_I,Est_ and *FWSI*_Ν,Est_, respectively.

On the other hand, it is well established that stomata of apples become closed lenticels with progressing fruit development, thus affecting transpiration rate of apple fruit [74–76]. The normalized *FWSI*_N,Est_ appeared lower at the first measuring date compared to later measurements and may, therefore, reflect such development, resulting in enhanced *FWSI*_N,Est_ values when transpiration rate was limited during fruit ripening (Fig. 4B). Again, the results provide a first view on the seasonal development of the index.

The relationship between Δ*T* and *FWSI* was not studied before. However, the difference between canopy temperature and *T*_a_ and its relationship with *VPD* has been investigated in several perennial trees. Enhanced and moderate correlations were observed in peach, nectarine, and orange, resulting in *r*^2^ of 0.70, 0.65, and 0.5, respectively [27,31,73]. In high-density olive orchards, *r*^2^ varied from 0.56 to 0.82 over the growing period [77]. Consistent with the previous observations made in several orchards that were adequately irrigated, no temperature difference or slightly cooler canopy temperature occurred considering measurements over several days [31,73,78]. Expanding data collection across different seasons and climatic conditions could provide a more comprehensive understanding of these dynamics, exploring the relationship between environmental factors and the *FWSI*_Est_ behavior.

### Diel course of *FWSI*

**Fig. 6.**
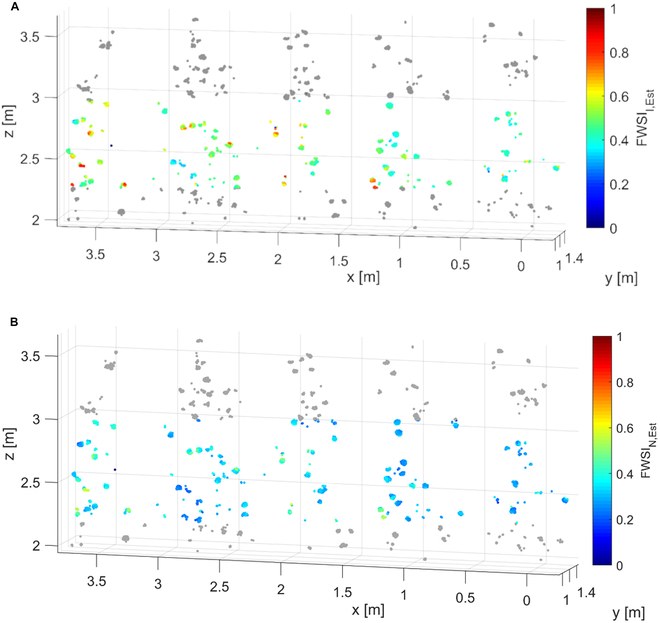
Spatial distribution of (A) *FWSI*_I,Est_ and (B) *FWSI*_N,Est_ within apple trees. Fruit temperatures necessary to estimate *FWSI* were measured remotely by means of *T*-annotated point clouds, presenting an example point cloud measured around noon of *DAFB*_153_.

Diel course was measured on ripe fruit during the commercial harvest window of apples over 2 consecutive days. The high resolution of LiDAR sensor allowed the spatial analysis of the variability of *FWSI* within the tree canopy (Fig. 6). The *FWSI* of apples declined in upper parts of canopy, with highest values found between 2.3 and 3 m. In this parts of canopy, *FWSI*_I,Est_ ranged between 0.4 and 0.8 (Fig. 6A), while values of *FWSI*_N,Est_ (Fig. 6B) spanned a slightly lower range (0.4 to 0.6). This finding needs to be evaluated further, as it may be due to methodological challenges, when measuring fruit slightly moving due to wind, and resulting lower temperature, when an object in the back or the sky was measured in the mixed pixel. As an additional factor, crop load influences the stomatal conductance of apple leaves, which is decreased by a reduction in fruit number [79]. For example, enhanced midday leaf *CWSI* values were observed in apple trees with no crop load, resulting in a smaller difference in canopy and air temperature [78]. However, due to exposure to global radiation, this would not mean that such exposed fruit are necessarily less affected by heat damage. However, it highlights the necessity of understanding the local variation in water relationships within the canopy as changes can have substantial effect on fruit quality and yield.

**Fig. 7.**
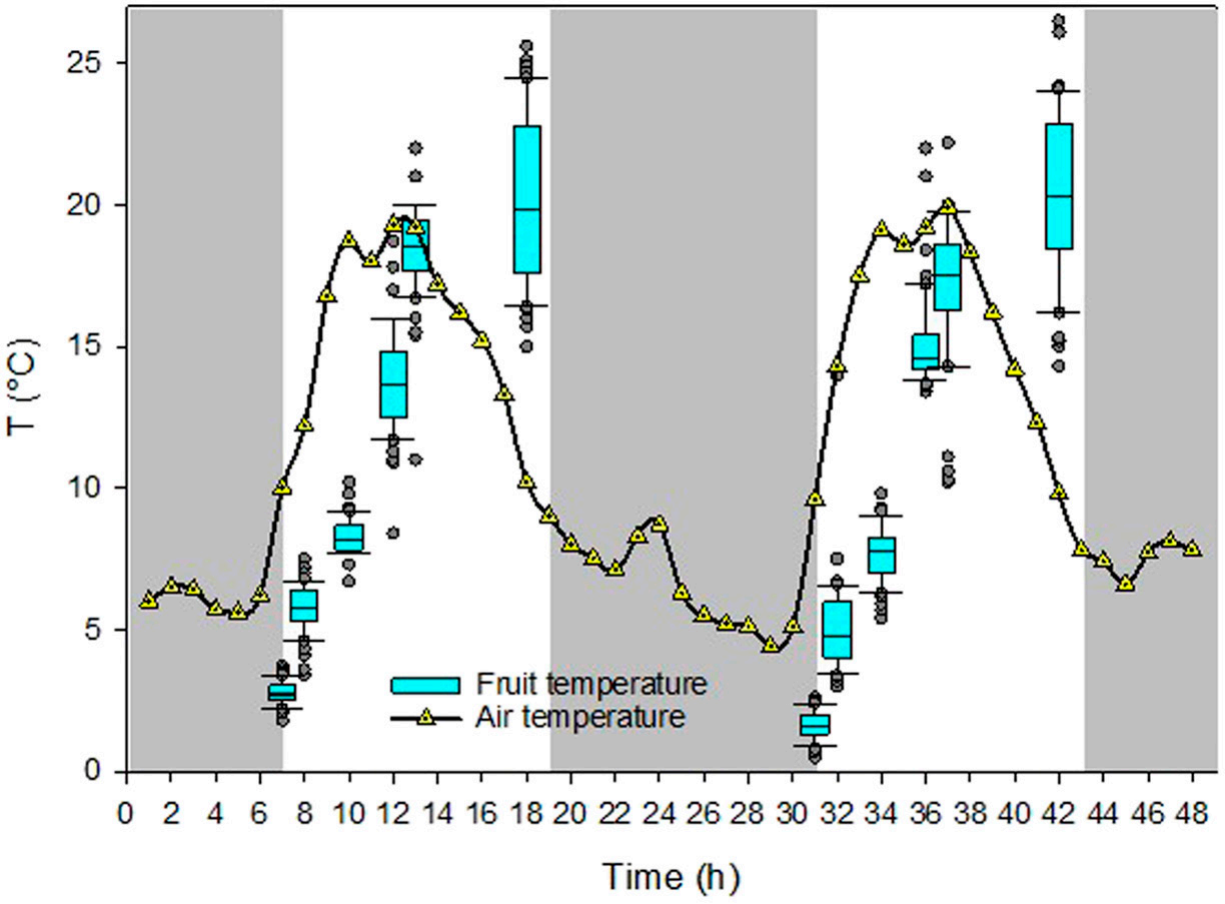
Diurnal courses of air (*T*_a_) and fruit surface temperature (*T*_Est_) measured on 2 successive *DAFB*_152_ and *DAFB*_153_ (September 21 and 22).

*CWSI* and *FWSI* showed no correlation (Fig. S3). The dataset acquired on last measuring date *DAFB*_166_ varied from other measuring dates, since the first 3 measurements during the season were taken at 1500, while the last one was conducted at 1300, when the fruits were still colder from the night time. Fruit temperature (*T*_Est_) followed the diurnal course of air temperature (*T*_a_) with a certain lag time (Fig. 7). On both measuring days, average *T*_a_ was approximately 23.5% and 28.6% higher during dawn (0700) compared to *T*_Est_. Moreover, variation of *T*_Est_ was low at this time, since fruit reached equilibrium with *T*_a_ during the night. The difference between *T*_a_ and *T*_Est_ was lowest around noon (1300). Only when *T*_a_ declined in the late afternoon (1800), fruit started to cool and Δ*T* became negative. Similar to fruit, temperature of leaves (*T*_Leaf_) was highest at 1800 on both days (Fig. S4A). At the same time, stem water potential (*Ψ*_Stem_) was lowest (Fig. S4B). Generally, variation of *Ψ*_Leaf_ increased after 11 h. A combination of midday stem water potential, predawn leaf water potential, and cumulative transpiration rate enabled the early prediction of water stressed in peach orchards [80].

**Fig. 8.**
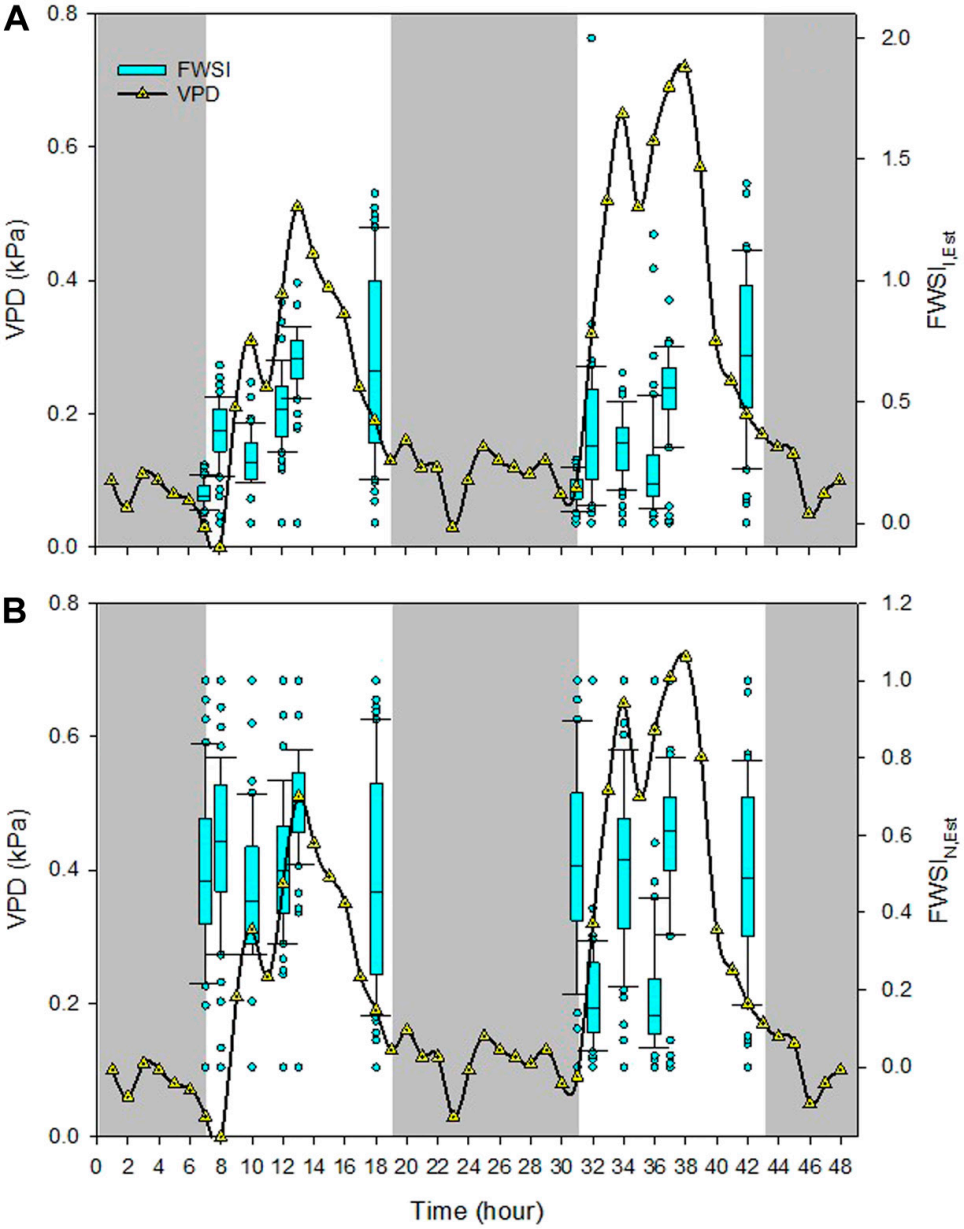
Diurnal courses of the water vapor pressure deficit (*VPD*) and *FWSI* calculated (A) according to *CWSI* proposed by Irmak et al. [64] (*FWSI*_I,Est_) and (B) by normalized index (*FWSI*_N,Est_).

Contrasting the strong dependence of *T*_Est_ from *T*_a_, the *FWSI* approaches appeared with different patterns. From 7 to 8 h, means of *FWSI*_I,Est_ rapidly increased from 0.15 (± 0.01) to 0.45 (± 0.03) and then only gradually to 0.62 (±) until 13 h (Fig. 8A). In the late afternoon (18 h), value and variation of *FWSI*_I,Est_ were high, neither following *T*_a_ nor *VPD* due to fruit mass and its temperature holding capacity serving as a buffer. A similar pattern was noticed on *DAFB*_153_. On that day, values of *FWSI*_I,Est_ again doubled within an hour, revealing 0.3 at 36 h and 0.6 at 37 h. On the other hand, means of *FWSI*_N,Est_ showed less pronounced differences between 7 and 8 h and throughout the day (Fig. 8B). Variation of *FWSI*_N,Est_ was only slightly enhanced on *DAFB*_153_, fluctuating between 0.14 and 0.62 without clear patterns.

So far, the principle of water-deficit stress indices was developed under the concept of the canopy temperature and leaf transpiration, which are sensitive to air temperature and *VPD*. With the present approach, the variation of fruit surface temperature can be monitored spatially with annotated point clouds, potentially providing new insight at the yield-relevant fruit scale. Similar approaches have been used to model fruit temperature at different locations [68], but relying on a model approach, while the method proposed in the present study would enable modeling work based on empirical analysis. However, the accuracy of noninvasive remote sensing techniques, such as LiDAR laser scanning in the field or orchard, is often compromised by occlusions and the presence of coinciding leaf surfaces. This issue is prevalent across various plant species as highlighted in recent studies by Deery et al. [46] and Keller et al. [81]. Therefore, potential differences between manual and LiDAR estimated parameters may appear to a varying extent in different tree architectures. Future studies should target the *FWSI* measurement in various production systems.

Overall, *FWSI* can be gained as a spatially and temporally resolved variable, enhancing the opportunities to analyze the fruit response to varying climate conditions. Furthermore, the efficiency and number of fruit analyzed could be enhanced by the remote sensing approach. This may increase the accuracy of analysis and modeling approaches through detailed spatiotemporal data.

The implementation of such remotely obtained plant data in existing agricultural systems can be streamlined due to its compatibility with remote sensing platforms currently developed for large-scale operations. This is crucial in large orchards and fields where manual monitoring of each individual plant or tree is impractical, while the low requirement for manual intervention makes it particularly suitable for extensive areas, where traditional monitoring methods may be less efficient or cost-prohibitive [82]. The application of *FWSI*_Est_ in real-world scenarios is supported by *CWSI* estimation at the canopy scale in pilot studies capturing several large orchards, but also by its feasibility in informing and optimizing irrigation scheduling [83]. Consequently, (i) more efficient water management practices should include *FWSI* at the crop scale to advance physiological models and (ii) also practical solutions can be effectively deployed in production systems based on the close-range remote sensing approach. Furthermore, the methodology can provide new insight in studies on sunburn damage [84].

## Conclusion

*T*-annotated 3D point clouds of fruit were acquired seasonally and in diel course under field conditions. Calibrated *T*_Est_ resulted in high coefficient of determination with reference measurements (*r*^2^ = 0.93). These thermal point clouds enabled the recording of spatiotemporally resolved water stress indices capturing leaves and newly introduced, considering segmented fruit data. The fruit water stress index showed reliable results, when calculated by means of air *T* plus 5 °C (*FWSI*_I,Est_). The diurnal course of fruit temperature evaluated from thermal point cloud followed the air temperature, revealing similar temperature at midday for 2 successive days. *FWSI*_I,Est_ provided patterns in both seasonal and diurnal courses. Overall, this study proves the applicability of thermal point cloud for *FWSI* analysis and the quantification of the temporal and spatial variation of this parameter. LiDAR-derived data on *FWSI* in combination with weather data and leaf or stem water potential may find application in irrigation scheduling and allow further ecophysiological studies on fruit response to varying climate conditions.

## Data Availability

Data underlying the results presented in this paper are available open access [62], with data in the supplement available from the authors upon request.
