# Supplementary material for: Fruit Water Stress Index of Apple Measured by Means of Temperature-Annotated 3D Point Cloud
Source: Plant Phenomics. 2024 Sep 18;6:0252. doi: 10.34133/plantphenomics.0252 (PMC11408935; doi:10.34133/plantphenomics.0252)
Supplement: Supplementary 1 — Figs. S1 to S6 Tables S1 and S2 [file plantphenomics.0252.f1.zip › Supplementary material.docx]

**Supplementary material**

(a)


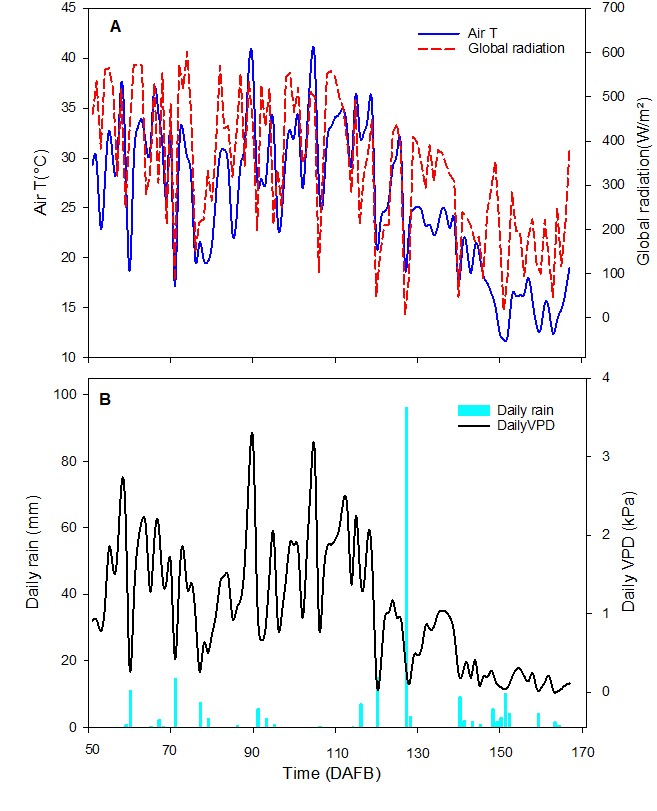


(b)

**Figure S1.** (**a**) Actual air temperature and global radiation as measured between days after and full bloom (*DAFB*) 50 to 170 at 15:00 h. (**b**) Values of daily precipitation and daily means of water vapor partial pressure deficit (*VPD*).

(a)


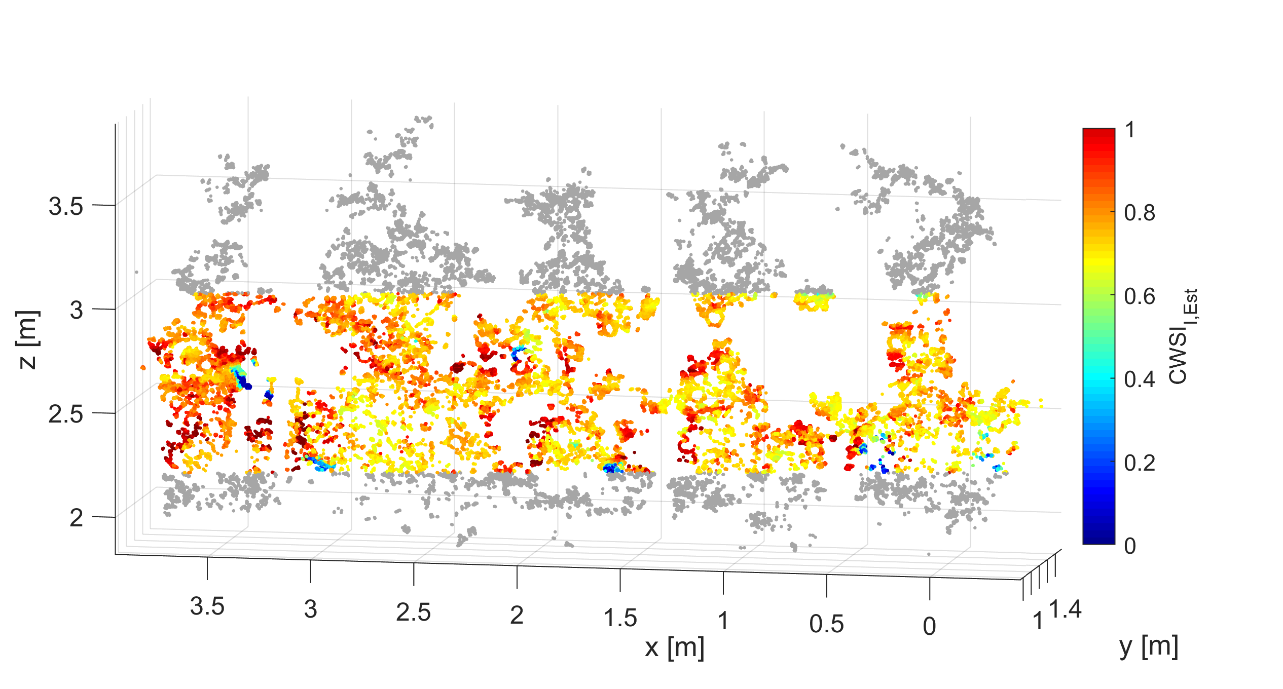


(b)


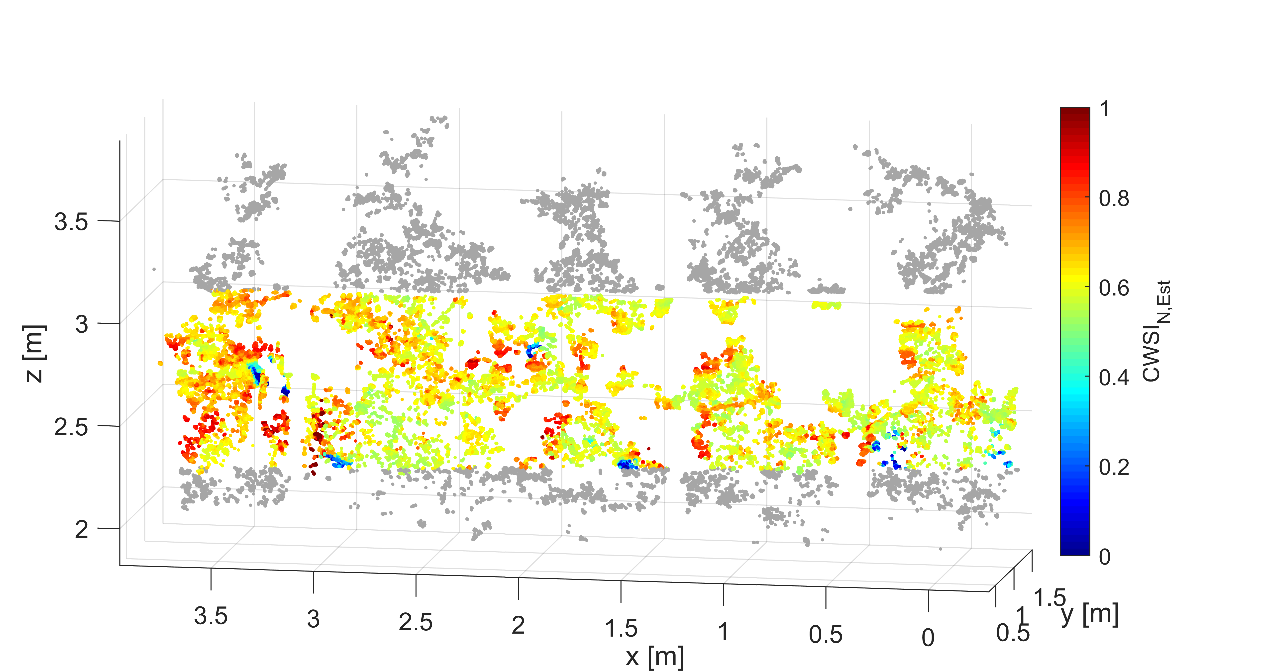


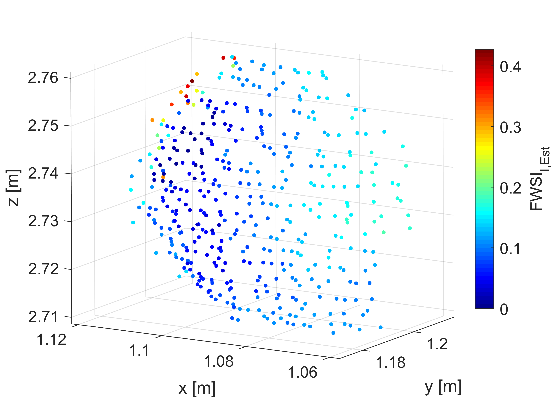

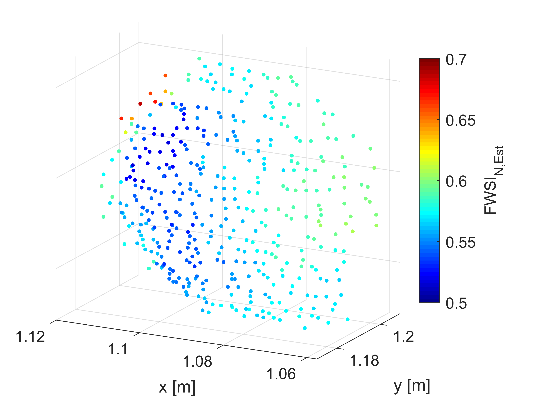


(d)

(c)

**Figure S2.** Point cloud of five canopies and example of segmented fruit point cloud considering (**a)** *CWSI*_I,Est_ and **(c**) *FWSI*_I,Est_ as well as (**b)** *CWSI*_N,Est_ and **(d**) *FWSI*_N,Est_ as measured by means of LiDAR and thermal sensors around noon on *DAFB*_153_.


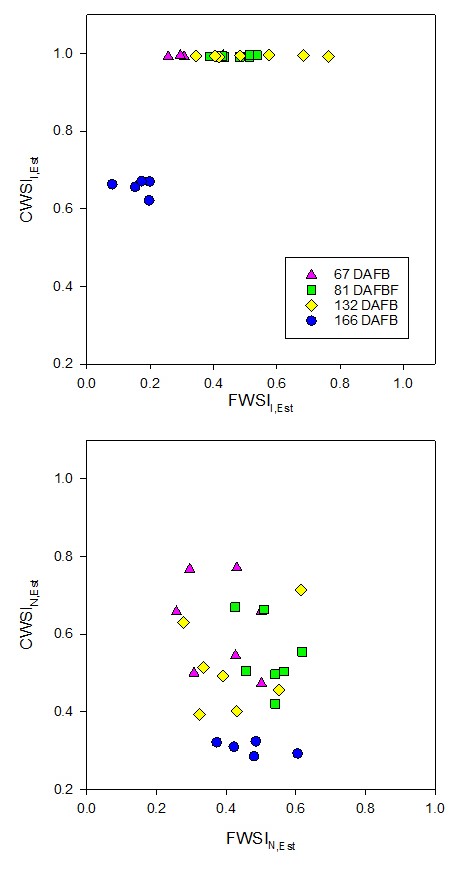

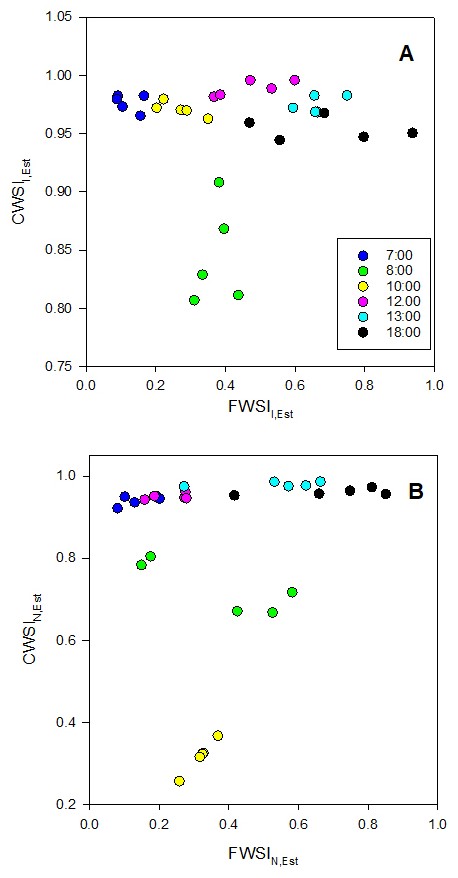

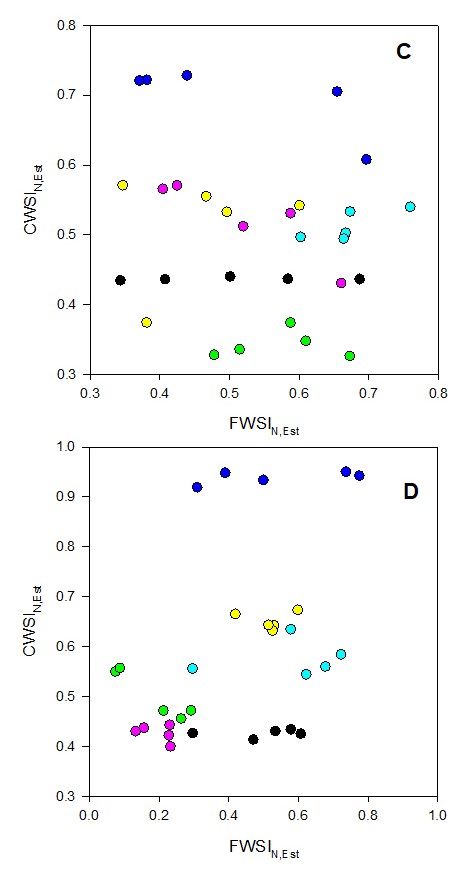


(f)

(e)

(d)

(c)

(b)

(a)

**Figure S3.** Relationship of crop water stress index (*CWSI*) and fruit water stress index (*FWSI*) measured (a, b) throughout the season in day after full bloom (DAFB) and as diel course (c, d) on 21^st^ September or (e, f) 22^nd^ September.


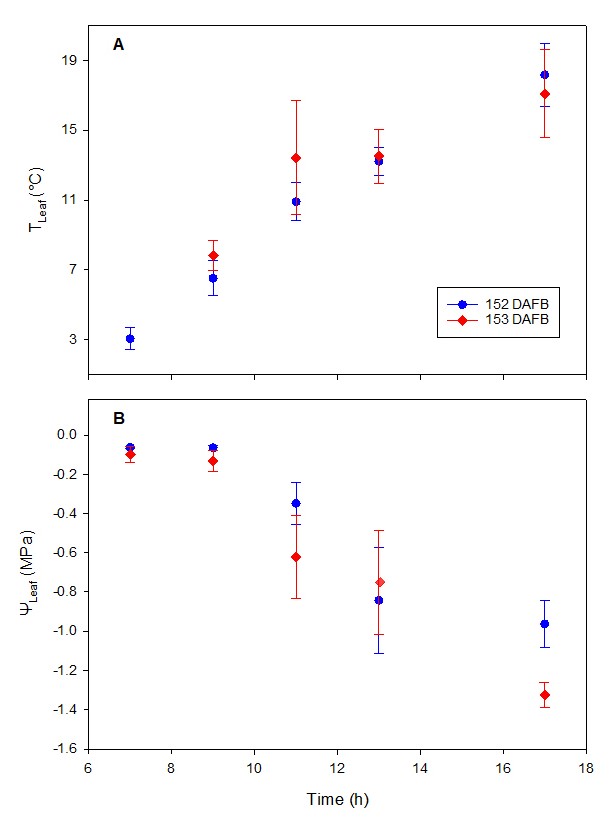


(b)

(a)

**Figure S4.** Diurnal changes of average (± SD; *n* =15) leaf temperature and stem water potential, as measured on two successive days (*DAFB*_152_ and *DAFB*_153_) on 21^st^ and 22^nd^ September, respectively.


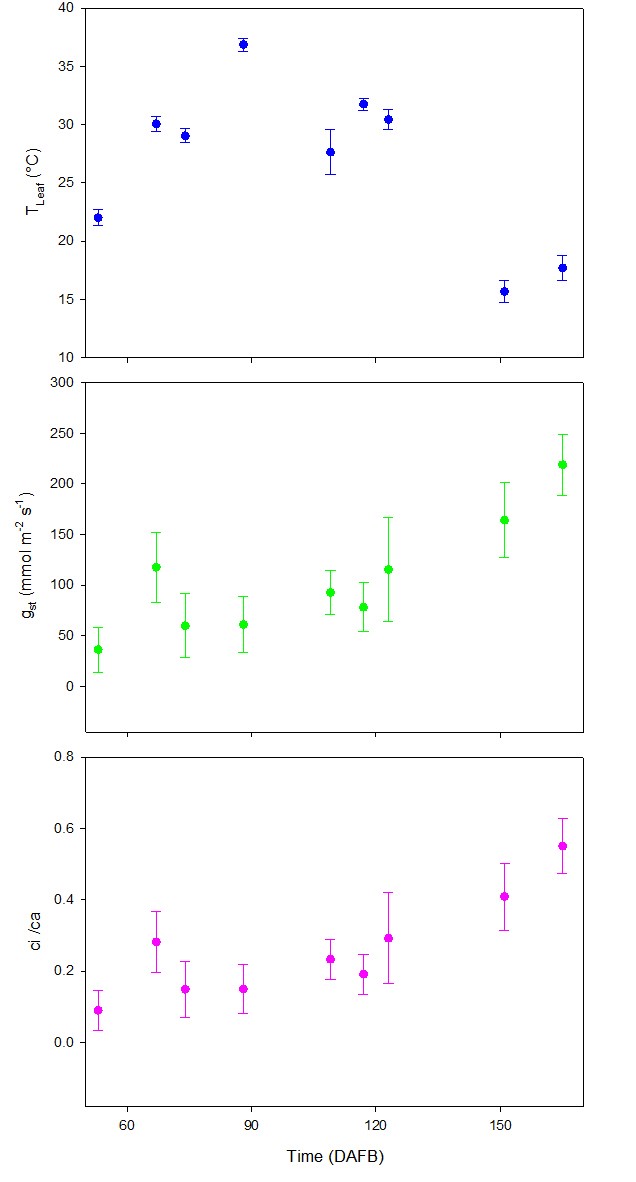


(c)

(b)

(a)

**Figure S5.** Means (± SD; *n* = 6 to 18) of leaf temperatures (*T*_Leaf_), stomatal conductance to water vapor transfer (*g*_st_) and the ratio of leaf internal and ambient CO_2_ concentrations (*c*_i_/*c*_a_) as measured during the course of the growing season on *DAFB*s 53, 67, 74, 88, 109, and 117 (*n* = 18) and 123, 151, and 165 (*n* = 12, *n* = 6 and *n* = 11, respectively).


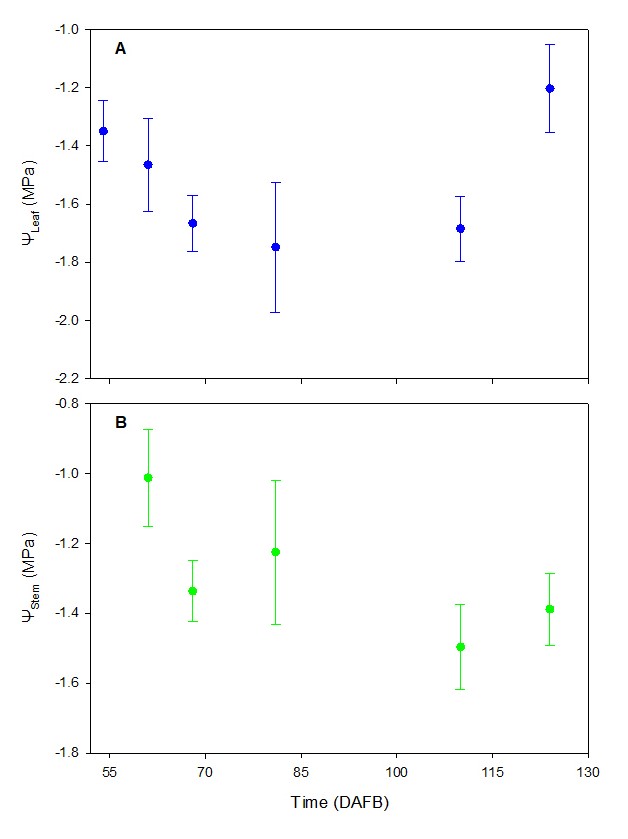


(a)

(b)

**Figure S6.** Means (± SD; *n* = 6) of leaf (a) and stem (b) water potential measured on various days after full bloom (*DAFB*_61_, *DAFB*_68_, *DAFB*_81_, *DAFB*_110_ and *DAFB*_124_) during the course of the growing season.

**Table S1.** Fruit quality (*n* = 6) as indicated by means (SD) of fruit fresh mass (*FM*), soluble solids content (*SSC*), fruit flesh firmness (^max^*F*) and dry mass related total chlorophyll content (*Chl*). Samples were taken on various days after full bloom (*DAFB).*

| *DAFB* | *FM* (g) | *SSC* (%) | ^max^*F* (N) | *Chl* (mg 100g^-1^) |
| --- | --- | --- | --- | --- |
| 67 | 31.4 (10.5) | 8.7 (0.8) | 182.1 (12.2) | 38.2 (9.6) |
| 81 | 47.5 (11.9) | 9.3 (0.6) | 157.6 (13.1) | 28.4 (5.0) |
| 132 | 105.7 (23.4) | 10.9 (1.1) | 103.7 (13.5) | 13.4 (4.2) |
| 166 | 150.4 (3.0) | 13.2 (1.3) | 77.2 (9.0) | NA |

**Table S2.** Stem dendrometer data (*n* = 2) considering the hysteresis height and fruit water stress index (*FWSI*) on different days after full bloom (*DAFB).*

| *DAFB* | *FWSI*_I,Est_ | *FWSI*_N,Est_ | Hysteresis height |
| --- | --- | --- | --- |
| 67 | 0.57 | 0.36 | 0.72 |
| 81 | 0.51 | 0.53 | 0.90 |
| 132 | 0.54 | 0.44 | 0.73 |
| 166 | 0.18 | 0.44 | 0.35 |
