# Supplementary figures and images for: Fruit Water Stress Index of Apple Measured by Means of Temperature-Annotated 3D Point Cloud
Source: Plant Phenomics. 2024 Sep 18;6:0252. doi: 10.34133/plantphenomics.0252 (PMC11408935; doi:10.34133/plantphenomics.0252)

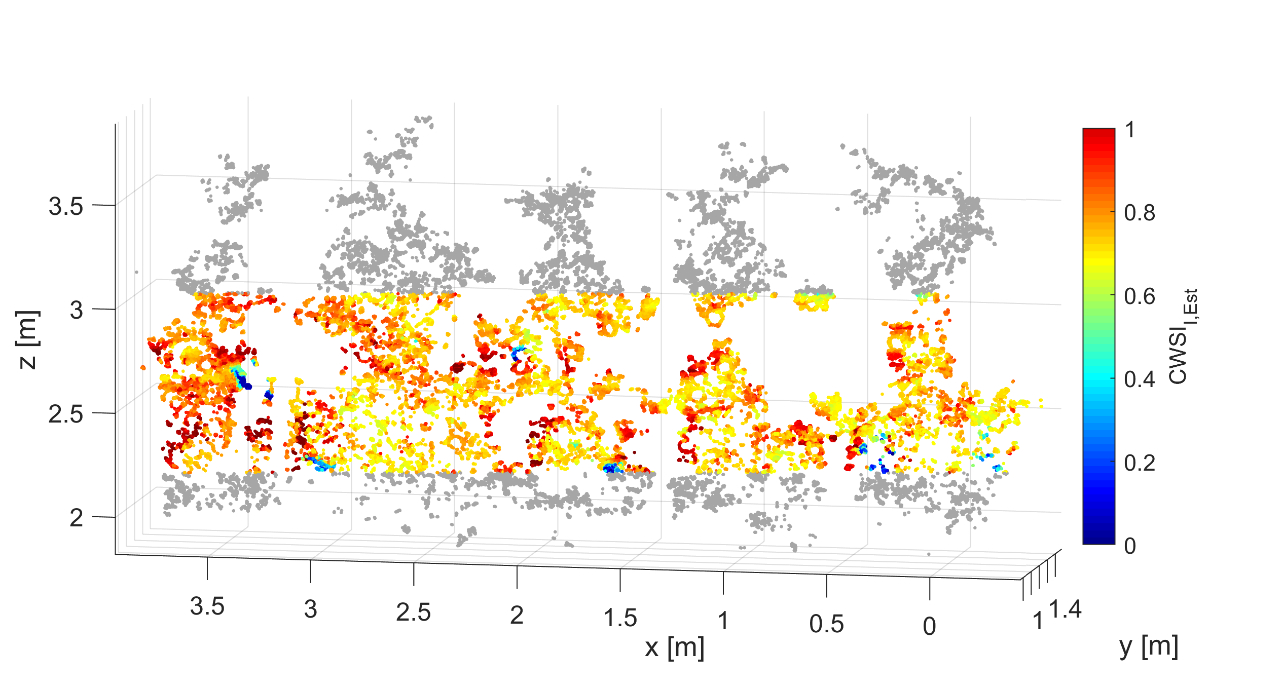

Supplement: Supplementary 1 — Figs. S1 to S6 Tables S1 and S2 [file plantphenomics.0252.f1.zip › FigureS2a.jpg]

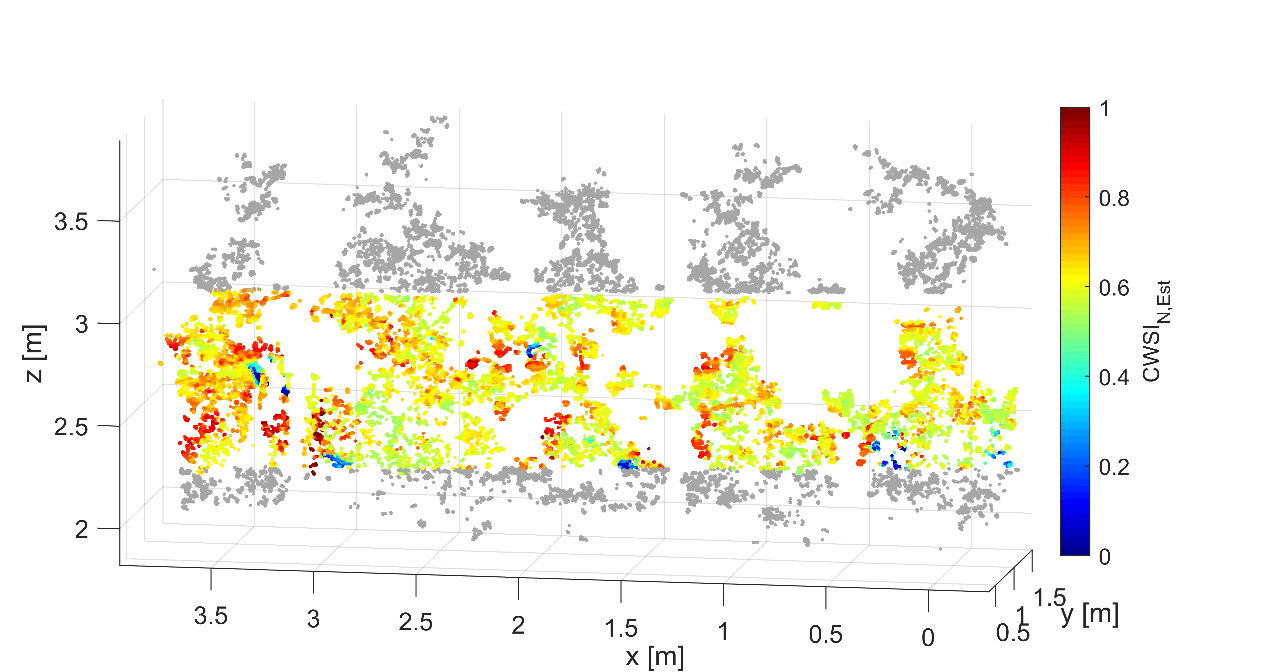

Supplement: Supplementary 1 — Figs. S1 to S6 Tables S1 and S2 [file plantphenomics.0252.f1.zip › FigureS2b.jpg]

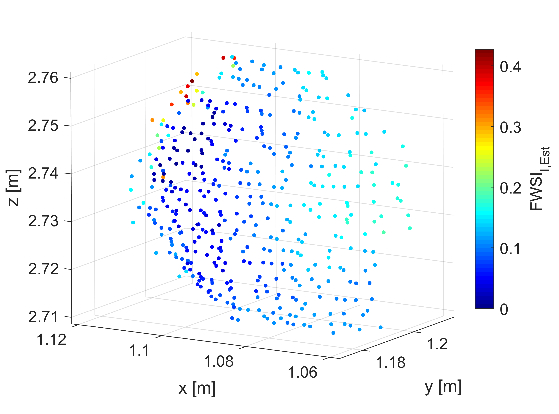

Supplement: Supplementary 1 — Figs. S1 to S6 Tables S1 and S2 [file plantphenomics.0252.f1.zip › FigureS2c.jpg]

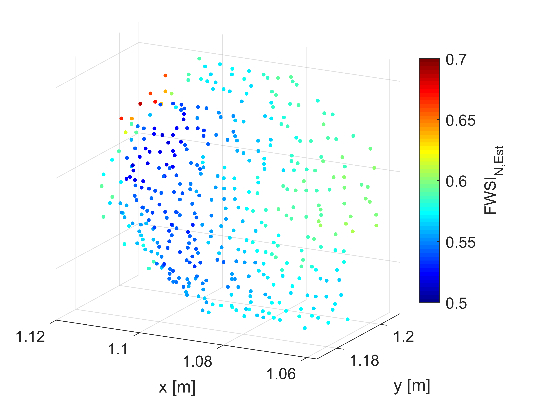

Supplement: Supplementary 1 — Figs. S1 to S6 Tables S1 and S2 [file plantphenomics.0252.f1.zip › FigureS2d.jpg]

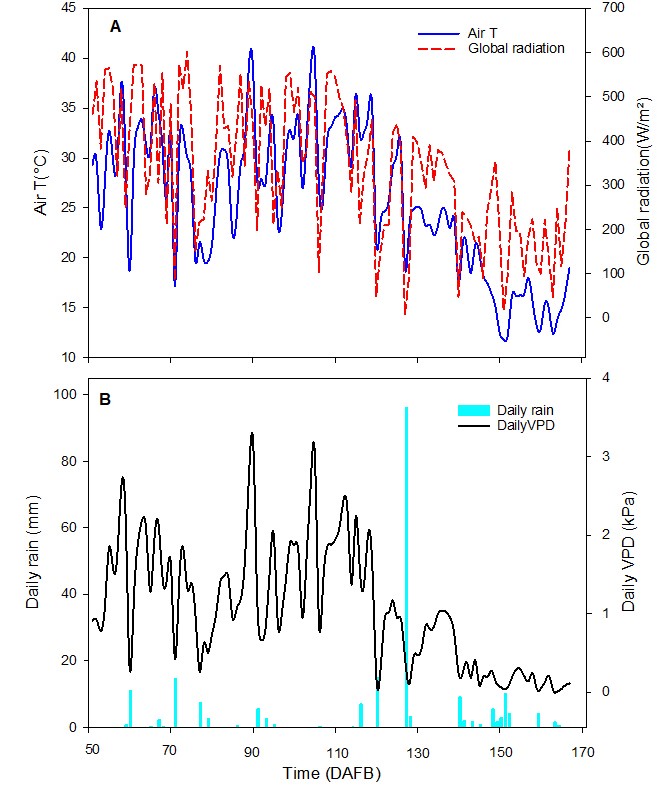

Supplement: Supplementary 1 — Figs. S1 to S6 Tables S1 and S2 [file plantphenomics.0252.f1.zip › s1.JPG]

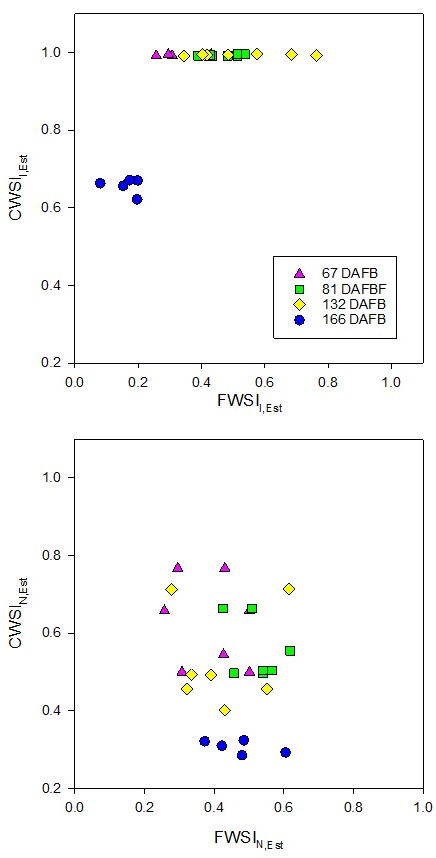

Supplement: Supplementary 1 — Figs. S1 to S6 Tables S1 and S2 [file plantphenomics.0252.f1.zip › s3-1.JPG]

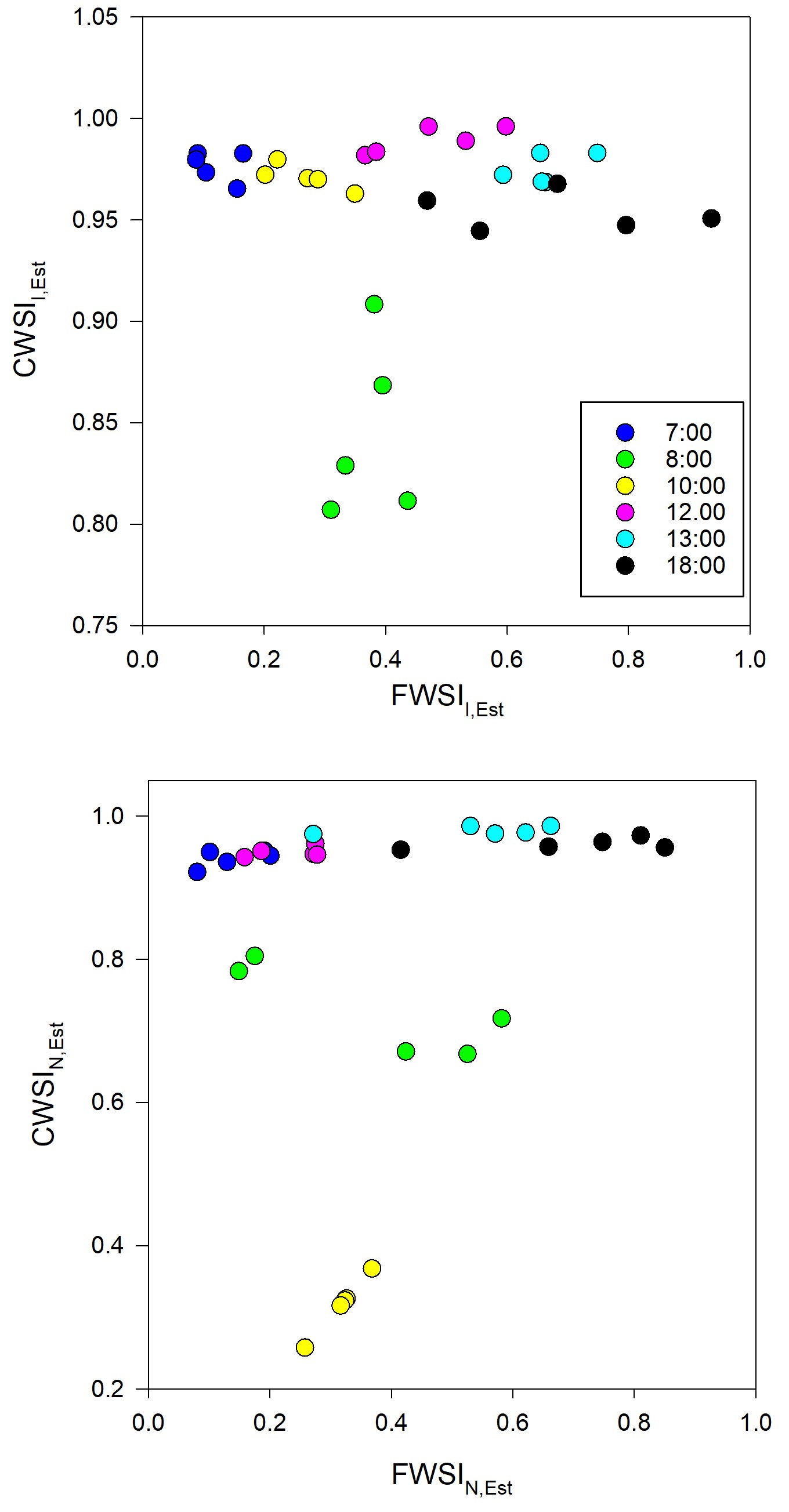

Supplement: Supplementary 1 — Figs. S1 to S6 Tables S1 and S2 [file plantphenomics.0252.f1.zip › s3-2.JPG]

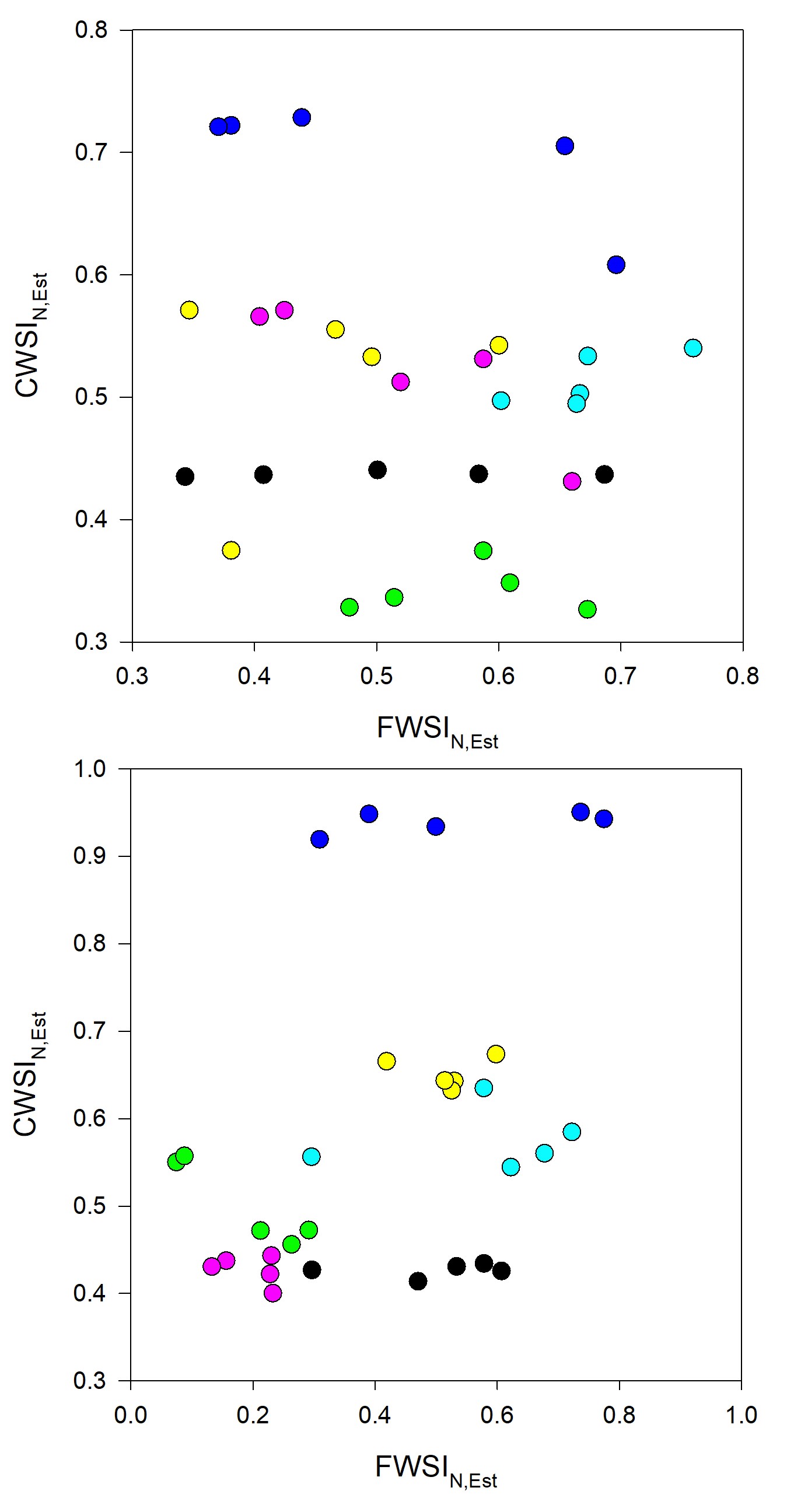

Supplement: Supplementary 1 — Figs. S1 to S6 Tables S1 and S2 [file plantphenomics.0252.f1.zip › s3-3.JPG]

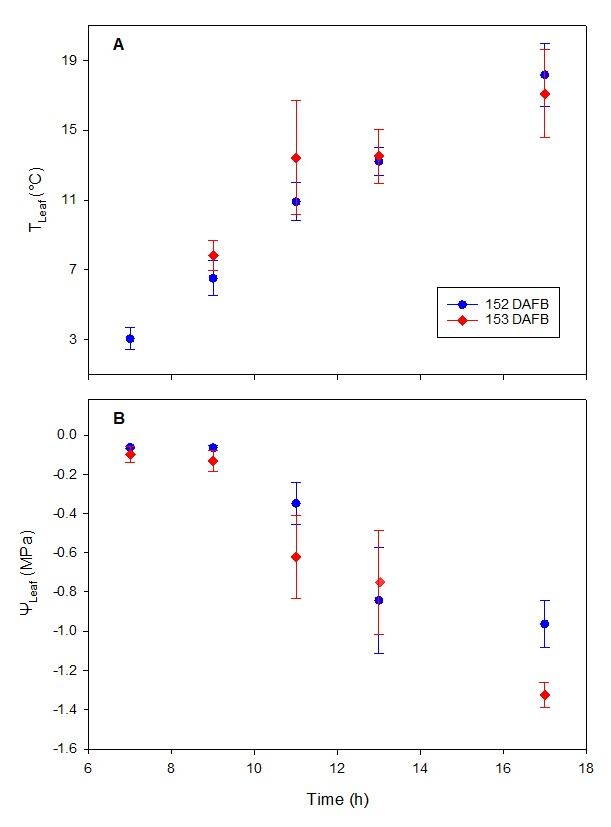

Supplement: Supplementary 1 — Figs. S1 to S6 Tables S1 and S2 [file plantphenomics.0252.f1.zip › s4.JPG]

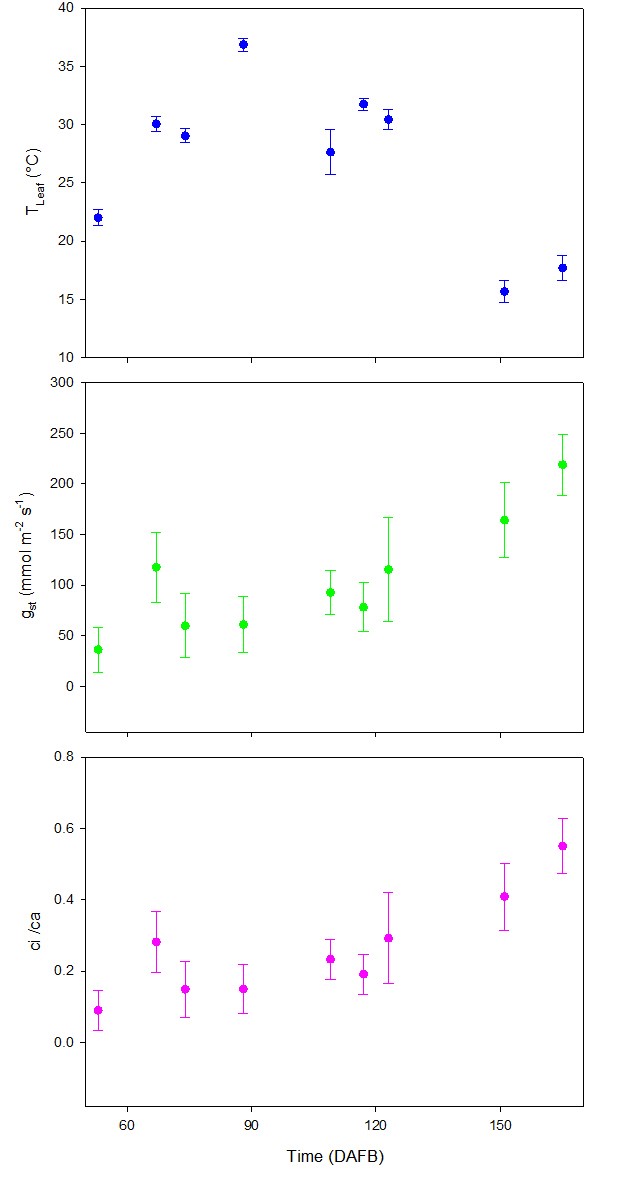

Supplement: Supplementary 1 — Figs. S1 to S6 Tables S1 and S2 [file plantphenomics.0252.f1.zip › s5.JPG]

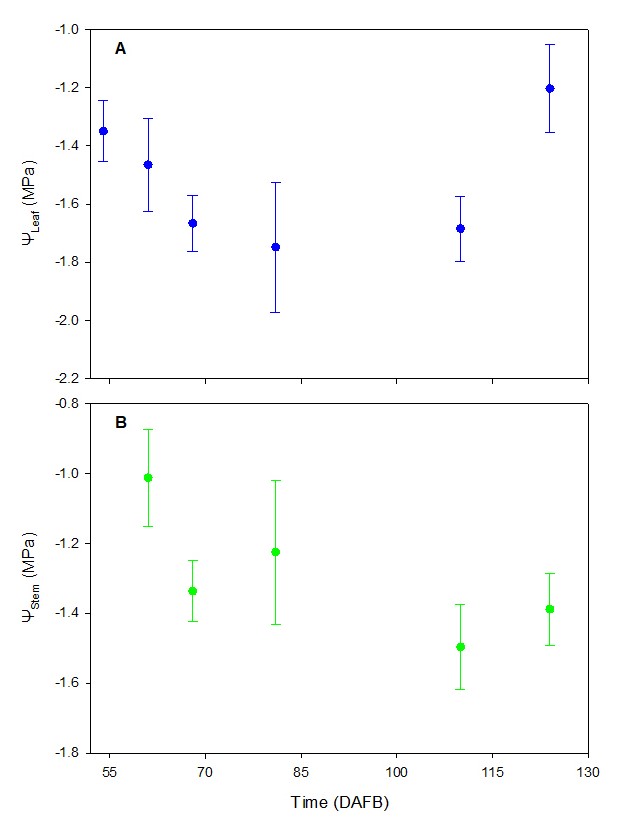

Supplement: Supplementary 1 — Figs. S1 to S6 Tables S1 and S2 [file plantphenomics.0252.f1.zip › s6.JPG]
